# A mitochondria-anchored supramolecular photosensitizer as a pyroptosis inducer for potent photodynamic therapy and enhanced antitumor immunity

**DOI:** 10.1186/s12951-022-01719-9

**Published:** 2022-12-03

**Authors:** Hong Wang, Guoxin Jing, Jintong Niu, Li Yang, Youyuan Li, Yi Gao, Huichao Wang, Xiaorong Xu, Yechang Qian, Shilong Wang

**Affiliations:** 1 Research Center for Translational Medicine at East Hospital, School of Life Science and Technology, Tongji University, Shanghai, 200092 People’s Republic of China; 2 Department of Respiratory Disease, Baoshan District Hospital of Integrated Traditional Chinese and Western Medicine, Shanghai, 201900 People’s Republic of China; 3 Department of Gastroenterology, School of Medicine, Shanghai Tenth People’s Hospital, Tongji University, Shanghai, 200072 People’s Republic of China

**Keywords:** Photodynamic therapy, Layered double hydroxides, Pyroptosis, Mitochondria anchoring

## Abstract

**Background:**

The discovery of a potent photosensitizer with desirable immunogenic cell death (ICD) ability can prominently enhance antitumor immunity in photodynamic therapy (PDT). However, majority of commercially-available photosensitizers suffer from serious aggregation and fail to elicit sufficient ICD. Pyroptosis as a newly identified pattern for potent ICD generation is rarely disclosed in reported photosensitizers. In addition, the photosensitizer with excellent mitochondria-anchored ability evokes prominent mitochondria oxidative stress, and consequently induces ICD.

**Results:**

Herein, a novel supramolecular photosensitizer LDH@ZnPc is reported, without complicated preparation, but reveals desirable pyroptosis-triggered ability with mitochondria anchoring feature. LDH@ZnPc is obtained through isolation of ZnPc using positive charged layered double hydroxides (LDH), and excellent mitochondria-anchored ability is achieved. More importantly, LDH@ZnPc-mediated PDT can effectively initiate gasdermin D (GSDMD)-dependent pyroptosis of tumor cells. In vitro and in vivo results verify robust ICD ability and potent tumor inhibition efficacy, and antitumor immunity towards distant tumor inhibition.

**Conclusions:**

This study reveals that LDH@ZnPc can act as an excellent pyroptosis inducer with simultaneous mitochondria anchoring ability for enhancing photodynamic therapy and boosting antitumor immunity.

**Graphical Abstract:**

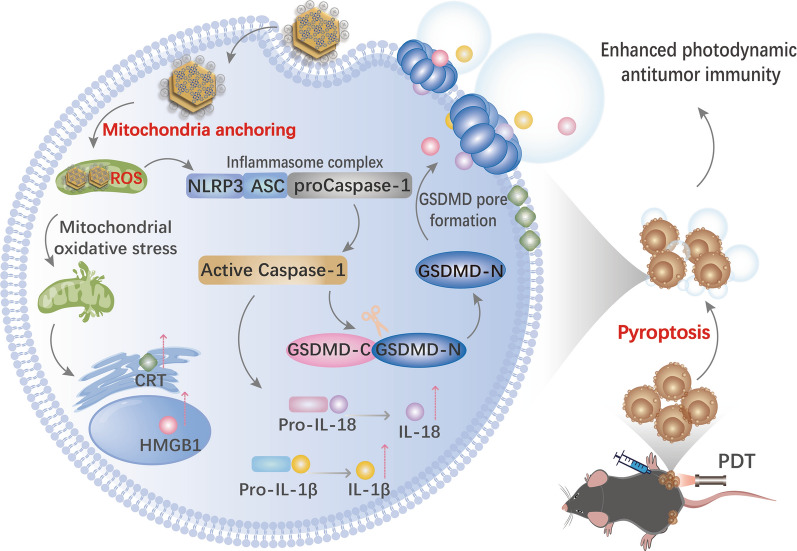

**Supplementary Information:**

The online version contains supplementary material available at 10.1186/s12951-022-01719-9.

## Introduction

Tumor immunotherapy as a highly effective treatment modality has recently attracted extensive attention [1]. Of note, immune checkpoint blockade (ICB), which uses small molecules or antibodies to block the immunosuppressive regulatory pathways, has revolutionized the therapies of several advanced cancers. However, ICB therapeutic effect is still far from desirable due to poor immunogenic response rates for many tumors [2, 3]. Consequently, it is imperative to develop an effective therapy with high efficacy as well as potential immunogenic induction for cancer treatment.

Recently, photodynamic therapy (PDT) as a non-invasive clinical procedure has attracted considerable attention, which has remarkable merits of overcoming drug resistance and reducing the side effects in treatment [4–6]. It uses light of a specific wavelength and photosensitizers to produce reactive oxygen species (ROS), particularly singlet oxygen (^1^O_2_), to destroy tumor tissues with highly temporal and spatial selectivity [7–9]. Particularly, much evidence has revealed that immunogenic cell death (ICD) was triggered after PDT, which can further enhance immunogenicity of cancer cells and prime subsequently adaptive immune response [10–12]. However, potent antitumor immune response caused by PDT is usually the exception instead of the rule [13–15]. Furthermore, improved antitumor immune effect was obtained in most cases wherein the photosensitizers combined with ICB inhibitors rather than used alone, which however increased the possibility of serious side effects [16].

Pyroptosis is a newly disclosed manner of immunogenic cell death, leading to the release of multiple pro-inflammatory cytokines and alarmins from dying cells, which then trigger robust antigen specific immune responses [17–19]. Gasdermin family proteins are key executors of pyroptosis, wherein the gasdermin D (GSDMD) is cleaved by Caspase-1 to form its N-terminal domain (GSDMD-N), which then binds with cellular membrane phospholipids and drills pores on cell membranes. As a consequence, the normal permeability of membrane is disrupted, thus leading to cell swelling and cell membrane rupture, followed by fast release of inflammatory related cellular contents (e.g., IL-1β, IL-18) [20, 21]. Very recently, Z. Liu et al. testified that the occurrence of pyroptosis of only minor tumor cells (less than 15%) is sufficient to clear the entire tumor graft [22]. Therefore, ICD induced by pyroptosis can trigger sufficient antitumor immunity, revealing potential novel therapeutic avenue to enhance the immunogenic of tumor cells. However, pyroptosis induction is currently limited to several chemotherapeutic drugs, while photosensitizers with pyroptosis initiation ability are rarely reported [14, 23, 24].

Currently, most of commercially available photosensitizers suffered from the robust tendency of aggregation in biological media [25–27]. Therefore, it is imperative to construct novel PDT modality with isolated photosensitizer in biological media and ability of triggering desirable immune response. Nanoscale layered double hydroxides (LDH) have recently shown great potential in biomedical applications [28–30]. With convenient synthesis, positive charged layered matrices and excellent biodegradability, LDH have been widely used in drug/gene delivery as well as inorganic-biology composites [31–38]. Phthalocyanines are representative type of photosensitizers due to their intense near-infrared absorption, potent efficacy and high singlet oxygen quantum yield [39, 40]. Previous researches have manifested that hydrophilic group modified phthalocyanines loaded by LDH achieved decreased aggregation and improved PDT efficacy, which provided a promising strategy to solve the widely existed dilemma of aggregation of photosensitizers [41–43].

Mitochondria have been demonstrated as a pivotal target in drug design of cancer and other diseases, particularly as a critical photooxidation target in PDT [44, 45]. As ROS possess very limited diffusion region (< 0.02 μm) and short half-life span (∼3.5 μs), the intracellular distribution of photosensitizers coincides with the photodamaged sites [46–48]. Therefore, photosensitizers capable of mitochondria localization ability actually enhance the efficiency of ROS, ultimately increasing the efficacy of PDT [27, 49, 50]. More interestingly, D. Ding et al. recently demonstrated that mitochondrial oxidative stress is an effective strategy to evoke robust ICD in tumors, revealing photosensitizer with mitochondria-anchored ability as a highly effective manner to enhance the immunogenicity of tumor cells [51]. In most cases, the achievement of mitochondria targeting relies on the positive charge of triphenylphosphonium moieties [52, 53]. Therefore, we can rationally infer that photosensitizer loaded by LDH, an intrinsic positive charged material, should also possess potent mitochondria targeting and ICD induction abilities, which have never been investigated within our scope of knowledge.

Encouraged by these aforementioned facts, we herein challenged a novel supramolecular photosensitizer via incorporation of zinc phthalocyanines (ZnPc) using LDH. The selection of ZnPc is mainly based on two concerns: (1) though ZnPc is extremely hydrophobic compared with hydrophilic groups modified phthalocyanines, it is much easier to chemical synthesize and purification, which is low-priced and convenient for large-scale production [39]; (2) the lyophilized liposomal formulation of ZnPc, namely CGP55847, was developed by Novartis and underwent phase I/II human clinical trials, which indicated that ZnPc is a promising photosensitizer for clinical candidate. However, CGP55847 still suffered from certain aggregation in biological media and thus limited its further investigation [54, 55]. To address aggregation-caused reduction of PDT efficacy, we take advantages of the layered matrices of LDH to sufficiently isolate hydrophobic ZnPc, thus preventing π–π stacking aromatic ring from aggregating. Furthermore, ZnPc was incorporated with positive charged LDH to obtain LDH@ZnPc, which possesses excellent mitochondria-anchored ability, hence making ROS generation mainly in mitochondria and then triggering mitochondrial oxidative stress, ultimately evoking efficent ICD generation [51]. More pleasantly, our results manifested that LDH@ZnPc can obviously evoke pyroptosis, which massively enhanced the ICD ability and hence potent antitumor immune response.

**Scheme 1:**
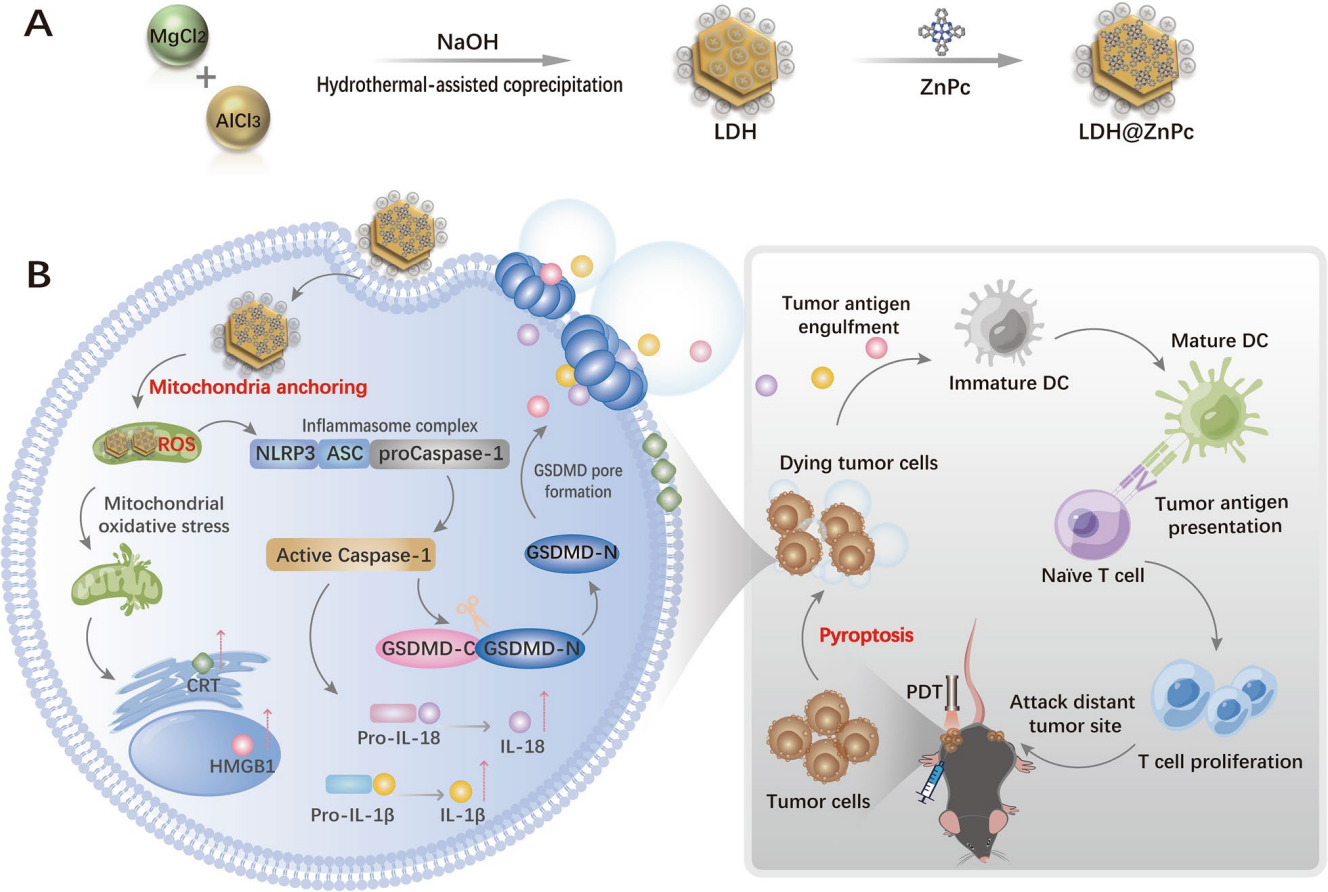
**A** Structure and fabrication of LDH@ZnPc. **B** Illustration of LDH@ZnPc featured with simultaneous mitochondria anchoring and pyroptosis abilities for potent photodynamic therapy and enhanced antitumor immunity

In this manuscript, the preparation of LDH@ZnPc, cellular toxicity, mitochondria localization, and ICD efficacy were carefully investigated. Furthermore, the cell morphology, mRNA and protein expression as well as transcriptomic analysis indicated the occurrence of GSDMD-dependent pyroptosis. In tumor-bearing C57BL/6 mice model, LDH@ZnPc-mediated PDT demonstrated robust antitumor effect, and potent immune inhibitory toward distant tumor. Besides, it apparently sensitized the therapy of αPD-1 when used simultaneously. Additionally, flow cytometry analysis of dendritic cells (DCs), CD4 + and CD8 + T cell levels further testified the excellent antitumor immune response via LDH@ZnPc-mediated PDT. Taken together, our supramolecular photosensitizer, without multiple complex incorporation system, can serve as an excellent pyroptosis inducer with simultaneous mitochondria anchoring ability for enhancing photodynamic therapy and boosting antitumor immunity (Scheme 1).

## Experimental section

### Chemicals and materials

Phosphate-Buffered Saline (PBS, pH 7.4), penicillin–streptomycin, and ROS detecting probe were purchased from Keygen (Nanjing, China). Fetal bovine serum (FBS), Dulbecco’s modified Eagle’s medium (DMEM), RPMI 1640, and trypsin were purchased from Thermo Fisher Scientific (MA, USA). Cell Counting Kit 8 (CCK-8), Annexin V-PI cell apoptosis kit, and JC-1 kit were purchased from Yeasen Biotechnology (Shanghai, China). Thiophene-2-thiol,4,6-dia-midino-2-10 phenylindole (DAPI), Mito-Tracker green, Golgi-Tracker green, ER-Tracker green, and Lyso-Tracker green were purchased from Beyotime (Shanghai, China). All fluorescent-labeled monoclonal antibodies were obtained from Biolegend (CA, USA) and Beyotime (Shanghai, China). αPD-1 antibody was supplied from BioXcell (NH, USA) and Zinc Phthalocyanine (ZnPc, 98%) was purchased from Adamas (Shanghai, China). All other chemical reagents were purchased commercially from Sinopharm Chemical Reagent Co., Ltd. (Shanghai, China).

### Cell culture

TC-1 (murine cervical cells) and HeLa (human cervical cells) were kindly provided by the Chinese Academy of Sciences (Shanghai, China). TC-1 cells and HeLa cells were cultured in DMEM and RPMI 1640 with 10% FBS containing penicillin/streptomycin (50 units mL^−1^) and 5% CO_2_ in an incubator at 37 °C.

### Animals

Inbred female C57BL/6 mice (6–8 weeks old) were purchased from the Shanghai Laboratory Animal Co., Ltd. (Shanghai, China) and housed in a specific pathogen-free (SPF) animal facility. All animal experiments were carried out according to the protocol approved by the Ethics Committee of Tongji University.

### Preparation of LDH and LDH@ZnPc

LDH nanoparticles were prepared by hydrothermal-assisted coprecipitation method according to the reported procedure with minor modification. Briefly, LDH was firstly precipitated by adding mixed salt solution (10 mL) containing MgCl_2_·6H_2_O (0.06 M) and AlCl_3_·9H_2_O (0.02 M) into NaOH solution (0.15 M, 40 mL) under nitrogen atmosphere and stirred vigorously for 30 min at room temperature. Subsequently, the resulting mixture was centrifuged at 5000 rpm for 5 min followed by removing of the supernatant, and then the sediment was dispersed into deionized water (40 mL) again. The procedures of centrifugation and dispersion were repeated twice. Finally, the obtained suspension was placed in an oven at 100 °C for 16 h. For the preparation of ZnPc-loaded LDH nanoparticles, the colloidally stable LDH nanoparticles were dispersed uniformly in DMSO (35 mL) and pH was adjusted to 9 by addition of triethylamine. Afterward, ZnPc (20 mg) was added to the above LDH solution and stirred at room temperature for 16 h. Finally, the LDH@ZnPc nanocomposites were collected by centrifugation (9000 rpm, 20 min), washed with DMSO, and finally rinsed with deionized water for three times.

### Characterization of LDH and LDH@ZnPc

Average diameter (Z-average), PDI, and zeta-potential quantification of LDH/LDH@ZnPc were conducted on Zetasizer Nano-SZ Instruments. All samples were dispersed in PBS and performed in triplicate. TEM imaging was obtained by a transmission electron microscope (JEOL, Japan) at 100 kV. SEM imaging assay was carried out using a transmission electron microscope (JSM-7800F, Japan). Elemental mapping of LDH@ZnPc was analyzed using a Thermo Scientific Talos F200X scanning transmission electron microscopy coupled with energy dispersive X-ray spectroscopy (STEM-EDS). Powder X-ray diffraction (PXRD) patterns of samples were collected at room temperature on a Rigaku Diffractomer Model Miniflex using CuKα radiation at 40 kV 30 mA, standard runs were performed with a scanning rate of 2°/min over a 2 range of 10–70°. X-ray photoelectron spectroscopy (XPS) was carried out on Thermo Kalpha XPS spectrometer using a monochromatic Al Kalpha source.

### Stability in storage and physiological conditions

LDH@ZnPc stability during storage was assessed by monitoring the change of absorbance during a period of 7 days. Aqueous suspensions were stored at 4 °C. In order to study stability in physiological conditions, LDH@ZnPc were incubated in PBS or FBS at 37 °C and stability was evaluated by motoring absorbance for 48 h.

### Drug loading and release of LDH@ZnPc

LDH@ZnPc nanocomposites of 2 mg dry powder was accurately weighed followed by addition of 200 μL HCl (1 M), and the obtained mixture was then sonicated for 20 min to dissolve the sample. The loading amount of ZnPc in LDH@ZnPc was determined by a 759S UV–Vis spectrophotometer (Lengguang Tech., China) at 670 nm and calculated using the following formulas:

DL (%) = (weight of ZnPc in LDH@ZnPc/weight of LDH@ZnPc) × 100%

For release investigation of LDH@ZnPc, 50 mg LDH@ZnPc were dispersed in 10 mL PBS (pH at 7.4, 6.5, 5.5, respectively) containing 0.1% Tween 20, which was transferred into a 14 kDa dialysis bag, followed by replacing in a flask filled with 100 mL PBS and incubating in a shaking air bath (37 °C, 120 rpm). The cumulative amount of ZnPc released into the medium was measured at predetermined time intervals using UV–Vis spectrophotometer at 670 nm.

### Cellular uptake

TC-1 cells were seeded on glass bottom dishes (Cellvis, D35-20–1.5P) at a density of 8 × 10^4^ per dish for 24 h. Afterward, the cells were incubated with ZnPc and LDH@ZnPc (C_ZnPc_ = 160 ng mL^−1^) for different intervals, followed by loading with DAPI to stain cell nucleus. Finally, in vitro cell uptake efficiency was investigated by CLSM (Leica SP8, Germany) on TC-1 cells. The cellular uptake of ZnPc and LDH@ZnPc was reflected by detection of intrinsic fluorescence from ZnPc.

### Colocalization assay

TC-1 cells were seeded on 35 mm glass bottom dishes at a density of 8 × 10^4^ per dish for 24 h. After being incubated with LDH@ZnPc for 1 h or 24 h, the cells were washed once with PBS and incubated with Mito-Tracker green (100 nM), Golgi-Tracker green (0.3 mg mL^−1^), ER-Tracker green (2 μM), and Lyso-Tracker green (50 nM), respectively. After removing of the trackers-containing DMEM, cells were washed with PBS twice and refed with fresh DMEM, followed by imaging on CLSM. Colocalization curves were generated using software (Leica SP8, Germany). The scatter plots and Pearson’s coefficients R of colocalization were then calculated by “Colocalization Finder” plugin in Image J.

### Depolarization of mitochondria membrane potential

TC-1 cells were cultured in 35 mm glass bottom dishes at a density of 8 × 10^4^ per dish for 24 h, followed by incubation with LDH@ZnPc for another 24 h. After treatment, cells were irradiated with a light dose of 3 J cm^−2^ using LED light (670 nm, 10 mW cm^−2^). 1 h later, JC-1 work solution was added in each dish and incubated for 30 min. The cells were washed with PBS and observed on a CLSM directly.

### Intracellular ROS level

The intracellular ROS level induced by LDH@ZnPc-mediated PDT was assessed on TC-1 cells by flow cytometry (BD, USA). Cells were seeded on a twelve-well plate at a density of 5 × 10^4^ per well and cultured for 24 h. After being treated with ZnPc and LDH@ZnPc (C_ZnPc_ = 10, 40, 160 ng mL^−1^) for another 24 h, cells were washed twice and then incubated with H_2_DCFDA in DMEM without FBS (10 μM, 500 μL) for 30 min. Then, the cells were exchanged with fresh DMEM and irradiated with a light dose of 3 J cm^−2^ (670 nm, 10 mW cm^−2^) using LED light. The cells were washed with PBS for three times and then trypsinized, which was then collected and further measured using flow cytometry as the vendor’s protocol.

### Cell viability assay

The cytotoxicity of ZnPc and LDH@ZnPc was evaluated on TC-1 and HeLa cells using CCK-8 assay and flow cytometry. The cells were seeded on two 96-well plates at a density of 3 × 10^3^ per well and further cultured for 24 h. ZnPc or LDH@ZnPc was added to the wells at an equivalent ZnPc concentration of 5, 10, 20, 40, 80, 160 ng mL^−1^ and incubated for another 24 h. Subsequently, one of the plates was irradiated with a light dose of 3 J cm^−2^ using LED light (670 nm, 10 mW cm^−2^) for phototoxicity, while another plate was treated without irradiation for dark cytotoxicity. The cells on both plates were incubated for 16 h and then 10 μL CCK-8 solution was added to each well, which was further incubated for 2 h. Finally, the absorbance at 450 nm was determined by a SpectraMax M5 microplate reader (Molecular Devices, USA). For flow cytometry analysis, cells were seeded on six-well plates at a density of 8 × 10^4^ per well and cultured for 24 h. The cells were treated with ZnPc or LDH@ZnPc at an equivalent ZnPc concentration of 160 ng mL^−1^ and incubated for 24 h. Afterward, the cells were exposed to a light dose of 3 J cm^−2^ from LED light (670 nm, 10 mW cm^−2^), and further incubated for 16 h. The cells were trypsinized and stained with Annexin V-PI cell apoptosis kit according to the vendor’s recommendation for flow cytometry.

### PDT-mediated pyroptosis in vitro

Evaluation of pyroptosis after PDT treatment was investigated using CLSM. TC-1 cells were seeded on 35 mm glass bottom dishes at a density of 8 × 10^4^ per dish for 24 h. The treatment of cells was the same as in flow cytometry, except that the incubation time was 2 h after PDT, and various groups in the dark were treated without irradiation. After treatment, the cells were imaged on CLSM immediately. Besides, quantitative real-time PCR (qPCR) and western blot assay were performed to further ascertain the occurrence of pyroptosis and its mechanism after LDH@ZnPc-mediated PDT.

### ICD efficacy

Immunogenic cell death (ICD) of TC-1 cells induced by PDT treatment was evaluated using immunofluorescence staining. The adhered TC-1 cells on 35 mm diameter glass bottom dishes were incubated with ZnPc and LDH@ZnPc (C_ZnPc_ = 160 ng mL^−1^) for 24 h and irradiated with LED light (670 nm, 10 mW cm^−2^) for 5 min, followed by incubating for another 12 h. Afterward, the cells were washed with PBS twice, fixed with 4% paraformaldehyde, permeabilized with 0.5% Triton X-100, and then blocked with 5% BSA. After treatment, the cells were incubated with rabbit anti-CRT antibody (Beyotime, AF1666) or rabbit anti-HMGB1 antibody (Beyotime, AF0180) at 4 °C overnight. Subsequently, the cells were washed with PBS three times and incubated with Alexa Fluor 488-labeled goat anti-rabbit secondary antibody (Beyotime, A0423) at 25 °C for 1 h, respectively. Then, the cells were washed with PBS three times and observed under CLSM.

### In vivo fluorescence imaging and antitumor activity

The C57BL/6 mice were subcutaneously injected with 100 μL of cell suspension containing 1 × 10^6^ TC-1 cells into the right flank under anesthesia with isoflurane. To observe the retention and metabolism of ZnPc and LDH@ZnPc in tumors, fluorescence imaging after intratumoral injection was performed by an in vivo imaging system (PerkinElmer IVIS Lumina XRMS III, USA) when the tumor volume reached 50–100 mm^3^. For the investigation of PDT efficacy, mice with tumor volume between 50 mm^3^ and 100 mm^3^ were randomized into six groups (n = 5). ZnPc or LDH@ZnPc at an equivalent ZnPc dose of 0.3 mg kg^−1^ was injected intratumorally, while the control group was treated with PBS. After 24 h, the mice in PBS (+), ZnPc (+), and LDH@ZnPc (+) groups were anesthetized with isoflurane and irradiated once using a laser light at 660 nm with a light dose of 120 J cm^−2^ (light power: 200 mW cm^−2^), and the groups of PBS (−), ZnPc (−), and LDH@ZnPc (−) were treated without irradiation. Afterward, the long (*L*) and short (*W*) diameters of tumors were measured every day using a digital caliper, and tumor volumes (*V*) were calculated as the formula: *V* = (*W*^2^ × *L*)/2. Finally, the mice were sacrificed on day 22 and the tumors were weighed and photographed. Tumors and major organs were fixed for H&E or TUNEL staining.

### Primary and distant tumor inhibition

Bilateral tumor models in C57BL/6 mice were established to evaluate the abscopal antitumor efficacy. 1 × 10^6^ TC-1 cells were injected into the right flank subcutaneous tissues on day 0 and 8 × 10^5^ TC-1 cells were injected into the left flank subcutaneous tissues on day 6 to mimic primary and distant tumors. When the primary tumor reached 50–100 mm^3^ in volume, the mice were injected intratumorally with LDH@ZnPc at a dose of 0.3 mg kg^−1^ or PBS with equal volume. 24 h after injection, the mice in each group were anesthetized with isoflurane and the primary tumors were irradiated once using a laser light at 660 nm with a light dose of 120 J cm^−2^ (light power: 200 mW cm^−2^). Subsequently, αPD-1 antibodies were injected intraperitoneally once every 2 days at a dose of 100 µg per mouse. On day 23, the mice were euthanized and tumors were harvested, which were then fixed for immunohistochemical and immunofluorescence staining.

### RNA sequencing

On day 23, primary and distant tumor tissues were collected and then total RNA was extracted for RNA sequencing. Transcriptomic sequencing results was conducted on a DNBSEQ platform at BGI Genomics.

### Immune response in vivo

On day 15, spleen, primary and distant tumor tissues were harvested and cut up thoroughly. Then, the tumors were digested with the mixture of collagenase type I (0.05 mg mL^−1^, Sigma), collagenase type IV (0.05 mg mL^−1^, Sigma) for 1 h at 37 °C in water bath and ground by the end of a syringe. The suspensions of spleens and tumors were filtered through nylon mesh filters with sizes of 70 µm and 40 µm respectively and washed twice with PBS. The resulting cell suspension was adjusted to a cell density of 1 × 10^6^ cells mL^−1^ and further labeled with fluorochrome-conjugated antibody CD45 (Biolegend, 103112), CD4 (Biolegend, 100405), CD8 (Biolegend, 100707), CD80 (Biolegend, 104705) and CD86 (Biolegend, 159203) respectively following the manufacturer’s protocol. After treatments, the cells were washed with PBS twice and analyzed using flow cytometry.

### Quantitative real-time PCR analysis

Total RNA was extracted by RNAiso Plus (Takara, China) according to the manufacturer’s instruction. Then, the concentration of RNA was quantified using a NanoDrop (Thermo Fisher Scientific, USA) and cDNA was obtained via reverse transcription using the PrimeScript RT reagent kit (Takara). Quantitative real-time PCR (qPCR) analysis was conducted using a TB Green Premix Ex Taq II kit (Takara) on a LightCycler LC96 real-time PCR system (Roche). Primers sequences were shown in Additional file 1: Table S2.

### Western blot analysis

The total protein of tumors was extracted using a protein extraction kit (KeyGEN, China), and the concentration was measured by the BCA protein assay (KeyGEN, China). An aliquot of 50 μg total protein was mixed with a 5 × protein-loading buffer (KeyGEN, China) and boiled for 5 min. Then, protein (20 μg) was separated by 10% SDS–polyacrylamide gel electrophoresis and then transferred into PVDF membranes (EMD Millipore, USA) in an ice bath. After transfer, non-specific antibody binding was blocked by incubating with 5% BSA (Sigma-Aldrich) at room temperature for 1 h, and then incubated with anti-GSDMD (Abcam, ab219800), Caspase-1 (Abcam, ab179515) and anti-β-actin (Abcam, ab8226) antibodies at 4 °C overnight, followed by washing and exposure to the secondary antibody for 1 h at room temperature. The imaging was visualized with ImageQuant LAS4000 mini (GE Healthcare Life Science, USA).

### Statistical analysis

Data analyses were performed by Graph Pad 7 software and statistical significance was analyzed with Student’s *t*-test and one-way ANOVA. * indicates a *p* < 0.05, ** indicates a *p* < 0.01, *** indicates a *p* < 0.001, and **** indicates a *p* < 0.0001.

## Results and discussion

### Preparation and characterization of LDH@ZnPc

**Fig. 1:**
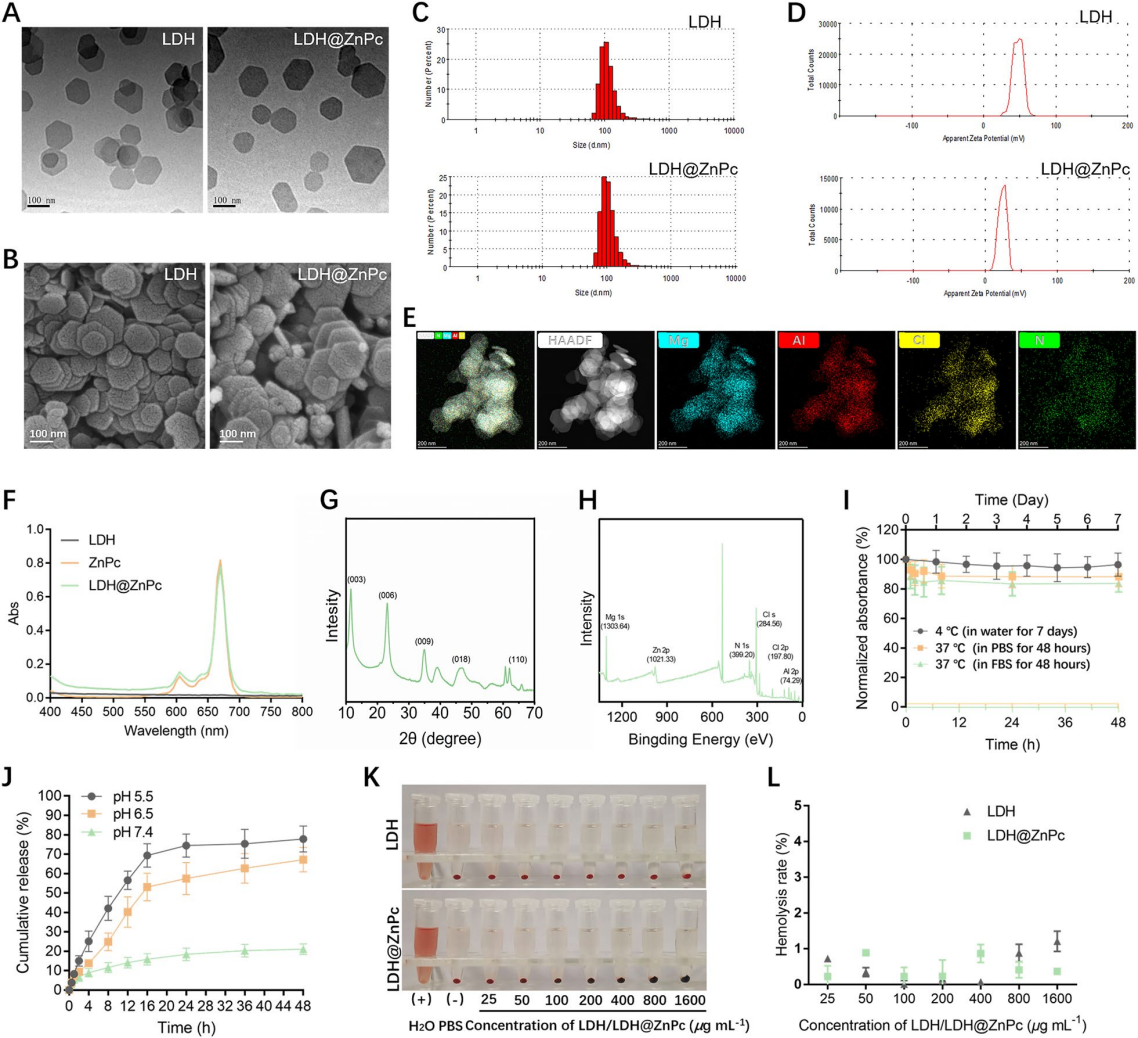
**A** TEM image of LDH and LDH@ZnPc. Scale bars: 100 nm. **B** SEM image of LDH and LDH@ZnPc. Scale bars: 100 nm. **C** Size distribution histogram of LDH and LDH@ZnPc. **D** Zeta potential of LDH and LDH@ZnPc. **E** Elemental mapping of LDH@ZnPc. Scale bar: 200 nm. **F** Absorption spectra of LDH, ZnPc and LDH@ZnPc. **G** XRD analysis of LDH@ZnPc. **H** XPS spectra of LDH@ZnPc. **I** Stability of LDH@ZnPC under storage (4 °C, in water) and physiological conditions (37 °C, in PBS and FBS). **J** In vitro ZnPc release from LDH@ZnPc. **K** Hemolysis assay of LDH and LDH@ZnPc and **L** the corresponding hemolysis percentages

LDH@ZnPc was synthesized through isolation of ZnPc by layered double hydroxides (LDH). Firstly, LDH nanoparticles with Mg^2+^ and Al^3+^ as metal cations, and chloride as interlayer anions were constructed by hydrothermal-assisted coprecipitation method. ZnPc was then incorporated onto the LDH with ZnPc loading of 3.17% ± 0.36% detected by UV − Vis spectrophotometer. The absorption spectrum of LDH@ZnPc was similar with that of ZnPc, indicating the integrity of ZnPc after incorporation by nonabsorbent LDH (Fig. 1F). Additionally, elemental mapping of LDH@ZnPc reveals that in addition to Mg, Al and Cl in LDH, N from ZnPc is also measured and uniformly distributed throughout the nanosheets of LDH, which well demonstrated the isolation of ZnPc using LDH (Fig. 1E). Transmission electron microscope (TEM) and scanning electron microscope (SEM) images showed that LDH possessed a uniform orthohexagonal morphology with particle size ranging from 80 to 150 nm, and LDH@ZnPc maintained the same morphology as LDH (Fig. 1A, B). Moreover, the equivalent hydrodynamic diameters of LDH and LDH@ZnPc in PBS were measured to be 120 ± 10.8 nm (polydispersity index, PDI = 0.13 ± 0.04) and 130 ± 18.2 nm (PDI = 0.19 ± 0.06) respectively (Fig. 1C), which are consistent with their corresponding images of TEM and SEM. The naked LDH displayed a highly positive zeta potential of + 44.1 ± 1.2 mV, while LDH@ZnPc still exhibited a relatively high value at + 23.9 ± 0.7 mV (Fig. 1D), which is sufficient to target mitochondria. The XRD patterns (Fig. 1G) of LDH@ZnPc showed the typical peaks (003), (006), (009), (018), and (110) of LDH, indicating that the incorporation of ZnPc did not affect the crystal structure of LDH. Besides, LDH@ZnPc elemental speciation was examined by XPS. There were six characteristic peaks in the XPS spectra of LDH@ZnPc (Fig. 1H), of which four of them were located at 1303.64, 74.29, 284.56, and 197.80 eV, which corresponded to Mg 1s, Al 2p, Cl s, and Cl 2p, respectively. We also observed two peaks at 1021.33 and 399.2 eV that corresponded with Zn 2p and N 1 s from ZnPc, providing important evidence that we successfully synthesized LDH@ZnPc.

The stability of LDH@ZnPc in storage condition and simulated physiological conditions was assessed by measuring the changes of absorbance. Figure 1I illustrates that LDH@ZnPc displayed negligible variation within 48 h of incubation in PBS or FBS, and that it remained substantially stable in water at 4 °C for 7 days. Moreover, a release pattern of the entrapped ZnPc from LDH@ZnPc formulation was observed in Fig. 1J. Only 20% of ZnPc was released from LDH@ZnPc after 48 h at pH value of 7.4. However, under pH of 6.5 and 5.5, LDH@ZnPc showed sustained drug release within 48 h. To further evaluate the blood safety of LDH and LDH@ZnPc, the hemolytic assay was conducted according to our previously reported method. When hemolysis occurs, the hemoglobin of RBCs will be released to make the solution turn red. As displayed in Fig. 1K, the control group treated with H_2_O exhibited robust red solution with no RBCs precipitated in the bottom, whereas the PBS-treated group as well as various concentration-treated groups of LDH and LDH@ZnPc revealed nearly red-free solution. Even the concentration of LDH and LDH@ZnPc was up to 1600 µg mL^−1^, no obvious hemolysis was observed. Meanwhile, the corresponding hemolysis rates were provided in Fig. 1L. Therefore, these results demonstrated LDH and LDH@ZnPc possess excellent in vitro blood safety.

### Cellular uptake and intracellular localization

**Fig. 2:**
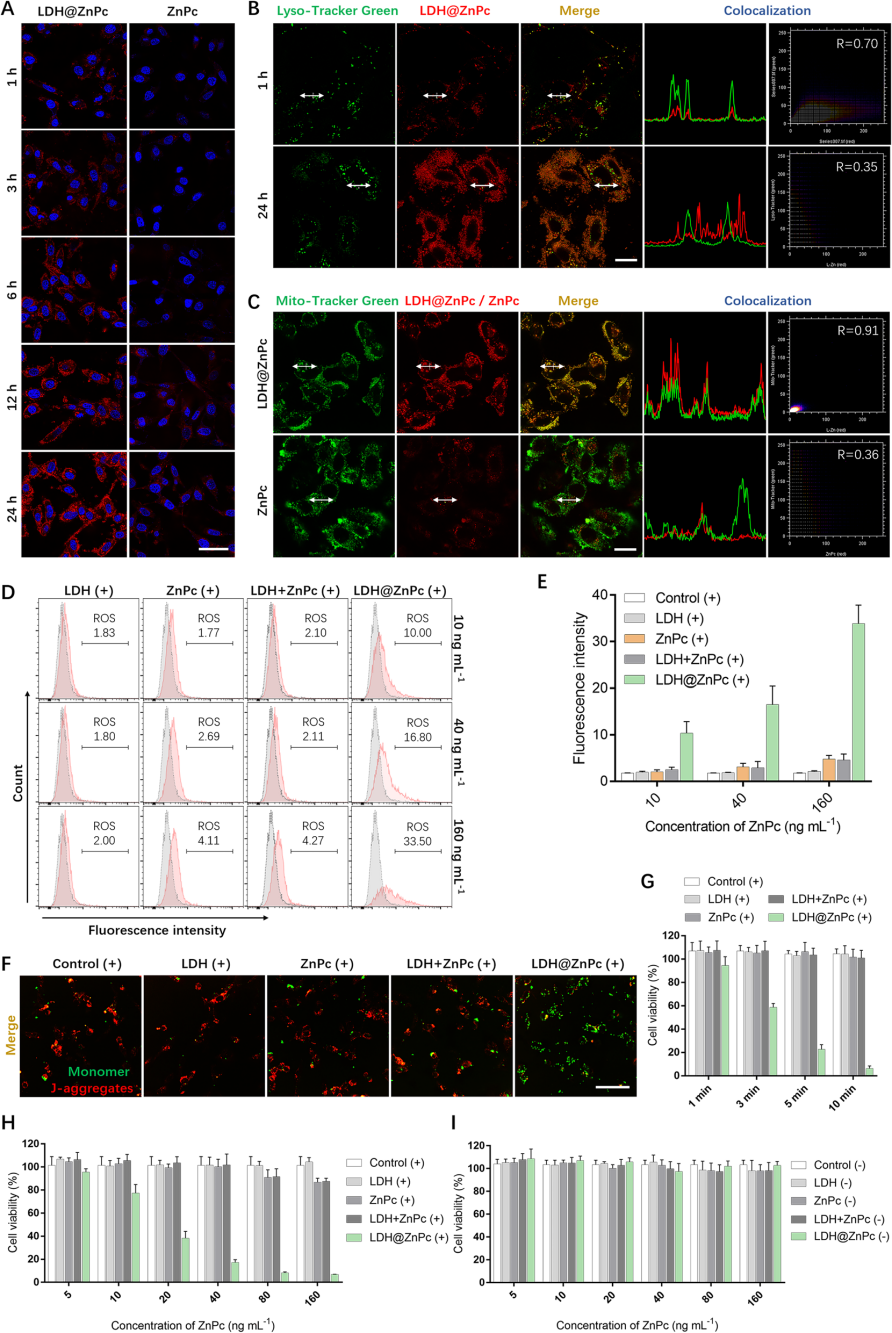
**A** Uptake determination of LDH@ZnPc and ZnPc at different incubation times. Scale bars: 50 µm. **B** Colocalization investigation of Lyso-Tracker Green and LDH@ZnPc at 1 h and 24 h. Scale bars: 20 µm. **C** Colocalization behavior between Mito-Tracker Green and ZnPc or LDH@ZnPc at 24 h. Scale bars: 20 µm. **D** ROS evaluation within TC-1 cells upon irradiation from LED light (670 nm, 3 J cm^−2^) of various groups at a dose of 10 ng mL^−1^, 40 ng mL^−1^ and 160 ng mL^−1^, respectively. **E** Mean fluorescence intensity of various groups in **D**. **F** Confocal fluorescence images reflecting the changes of mitochondria membrane potential in TC-1 cells using JC-1 dye as the indicator. Scale bar: 50 µm. **G** Evaluation of cell viability in TC-1 cells of various groups at multiple light irradiation times (670 nm, 10 mW cm^−2^). **H** Cell viability determination of various groups after irradiation from LED light (670 nm, 3 J cm.^−2^). **I** Cell viability determination of various groups in the dark. (“ + ” and “−” denote with and without irradiation, respectively)

We first investigated cellular uptake of LDH@ZnPc in TC-1 cells at various time by confocal laser scanning microscopic (CLSM). As shown in Fig. 2A and Additional file 1: Figure S1, LDH@ZnPc was rapidly internalized by TC-1 cells in 1 h and exhibited a much higher cellular uptake than ZnPc. In addition, fluorescence signals were barely observed in TC-1 cells incubated with ZnPc within 6 h. Overall, the fluorescence intensity exhibited a time-dependent manner within investigated times, and LDH@ZnPc displayed desirable intensity at 24 h, which is obviously stronger than that of ZnPc. These results indicated the efficient uptake of cationic LDH@ZnPc in TC-1 cells, which is consistent with our previous reports of cellular uptake using positively charged LDH [33].

The mitochondria-anchored ability of photosensitizers is a critical parameter evaluating the therapeutic efficacy of PDT [27, 50, 56]. Therefore, the subcellular organelle localization behavior of LDH@ZnPc was then investigated by CLSM and further quantified using Image J. As exhibited by the fluorescence in Fig. 2B, LDH@ZnPc was quickly taken up and trapped within 1 h in lysosomes. Notably, the fluorescence of LDH@ZnPc in lysosomes almost disappeared at 24 h (Fig. 2B), whereas the fluorescence in mitochondria enhanced significantly (Fig. 2C). Besides, the colocalization Pearson’s coefficients (R) between ZnPc and mitochondria was only 0.36, while the value between LDH@ZnPc and mitochondria was up to 0.91 (Fig. 2C). Furthermore, the colocalization behaviors of LDH@ZnPc with golgi apparatus and endoplasmic reticulum were also investigated. As reflected in Additional file 1: Figure S2, LDH@ZnPc overlapped partially with Golgi-Tracker and ER-Tracker with Pearson’s coefficients of 0.63 and 0.61, respectively, indicating that LDH@ZnPc did not have obvious preference on golgi apparatus and endoplasmic reticulum. Collectively, these results demonstrated the excellent mitochondria-anchored ability of LDH@ZnPc.

### ROS generation and depolarization of mitochondrial membrane potential

The produced ROS in PDT play a significant role in killing tumor cells, and therefore the intracellular ROS generation is closely associated with the therapeutic efficacy. To examine whether isolation of ZnPc with LDH can facilitate ROS generation, we then evaluated the intracellular ROS levels with various doses of LDH@ZnPc in TC-1 cells. 2', 7'-dichlorodihydrofluorescein diacetate (H_2_DCFDA) was used as ROS indicator, which can be deacetylated by endocellular esterases and further oxidized by generated ROS within cells. The oxidized product is retained intracellularly and thus can be monitored through detecting its green fluorescence (525 nm). As displayed in Fig. 2D, E, cells incubated with 10 ng mL^−1^ of ZnPc upon irradiation exhibited a ROS-positive cell population of merely 1.77%, which was similar with that of the control. In comparison, cells demonstrated significantly increased ROS levels upon irradiation with a LED light (670 nm, 3 J cm^−2^) in the presence of 10 ng mL^−1^, 40 ng mL^−1^, and 160 ng mL^−1^ of LDH@ZnPc, which demonstrated cell population percentages of 10.0%, 16.8%, and 33.5%, respectively. It is noteworthy that the prominent results were achieved under such an ultralow light dose of 3 J cm^−2^ in PDT, which well testified the potent phototoxicity of LDH@ZnPc. Besides, these results also indicated that LDH@ZnPc-mediated ROS generation displayed a dose-dependent pattern in TC-1 cells within the investigated concentration ranges.

The membrane potential of mitochondria is a critical indicator reflecting its normal function, and the potential can be disrupted when ROS-mediated PDT occurred in mitochondria. To investigate the change of mitochondria membrane potential, the assay was thus performed after PDT using JC-1 dye as the indicator. In energized mitochondria, JC-1 can accumulate in the matrix of mitochondria and form a polymer emitting strong red fluorescence (~ 590 nm). However, red JC-1 polymer transforms to green JC-1 monomer (~ 529 nm) when mitochondrial depolarization occurs. As shown in Fig. 2F and Additional file 1: Figure S3, the normal cells displayed strong red fluorescence, whereas LDH@ZnPc incubated cells showed intense green fluorescence upon irradiation with nearly no observed red light, indicating that membrane potential of mitochondria was severely destructed by LDH@ZnPc-mediated PDT.

### Cytotoxicity evaluation in vitro

Construction of supramolecular photosensitizer with high phototoxicity and simultaneously low dark toxicity is of great importance during PDT. Therefore, we further examined the cell toxicity of LDH@ZnPc. TC-1 cells were incubated with different dilutions of ZnPc and LDH@ZnPc followed by irradiation, and cell viability was evaluated by cell counting kit-8 (CCK-8) assay and flow cytometry assay, respectively. Meanwhile, the cellular dark toxicity was determined as well using parallel studies with no irradiation. As shown in Fig. 2H, after 24 h incubation of ZnPc, the viability of TC-1 cells remained normal, suggesting that only incubation with ZnPc was not sufficient to kill tumor cells after irradiation from LED light (670 nm, 3 J cm^−2^). In contrast, the phototoxicity of LDH@ZnPc was significantly enhanced after light irradiation, resulting in a concentration-dependent manner toward cell killing, which also supported mitochondria localization for enhanced therapeutic efficacy. In particular, the TC-1 cells were almost completely eradicated when the dose of LDH@ZnPc rose to 80 ng mL^−1^. On the contrary, negligible change in TC-1 cell viability was observed without LED light irradiation, which indicated that both LDH@ZnPc and ZnPc displayed negligible dark toxicity (Fig. 2I). Furthermore, the phototoxicity of LDH@ZnPc at various irradiation times was also performed in TC-1 cells, which exhibited a time-dependent manner within investigated time (Fig. 2G). To further testify the obtained results, flow cytometry assay was performed and confirmed the potent phototoxicity of LDH@ZnPc than ZnPc alone or a mixture of LDH and ZnPc (Additional file 1: Figure S4). Besides, we also investigated the phototoxicity behavior of our photosensitizer in HeLa cells, which displayed similar trends to that observed in TC-1 cells, with LDH@ZnPc showed the most potent efficacy (Additional file 1: Figure S5). Taken together, these results demonstrated that LDH@ZnPc presented high phototoxicity and low dark cytotoxicity within examined cells.

### PDT-induced pyroptosis in vitro

Photosensitizers with mitochondria-anchored ability can induce photodamage on mitochondria and the subsequently mitochondrial oxidative stress, disrupting its own structure and leading to changes in cell morphology [51]. We have demonstrated the severe destruction of mitochondrial membrane potential induced by LDH@ZnPc-mediated PDT. Herein, to provide more insight of mitochondria targeting photodynamic cytocidal action, the changes of TC-1 cell morphology were recorded by CLSM. After being cultured with photosensitizers and exposed to light irradiation (670 nm, 3 J cm^−2^) or in the absence of light, the TC-1 cells were cultured for another 2 h before the cell morphology was observed.

**Fig. 3:**
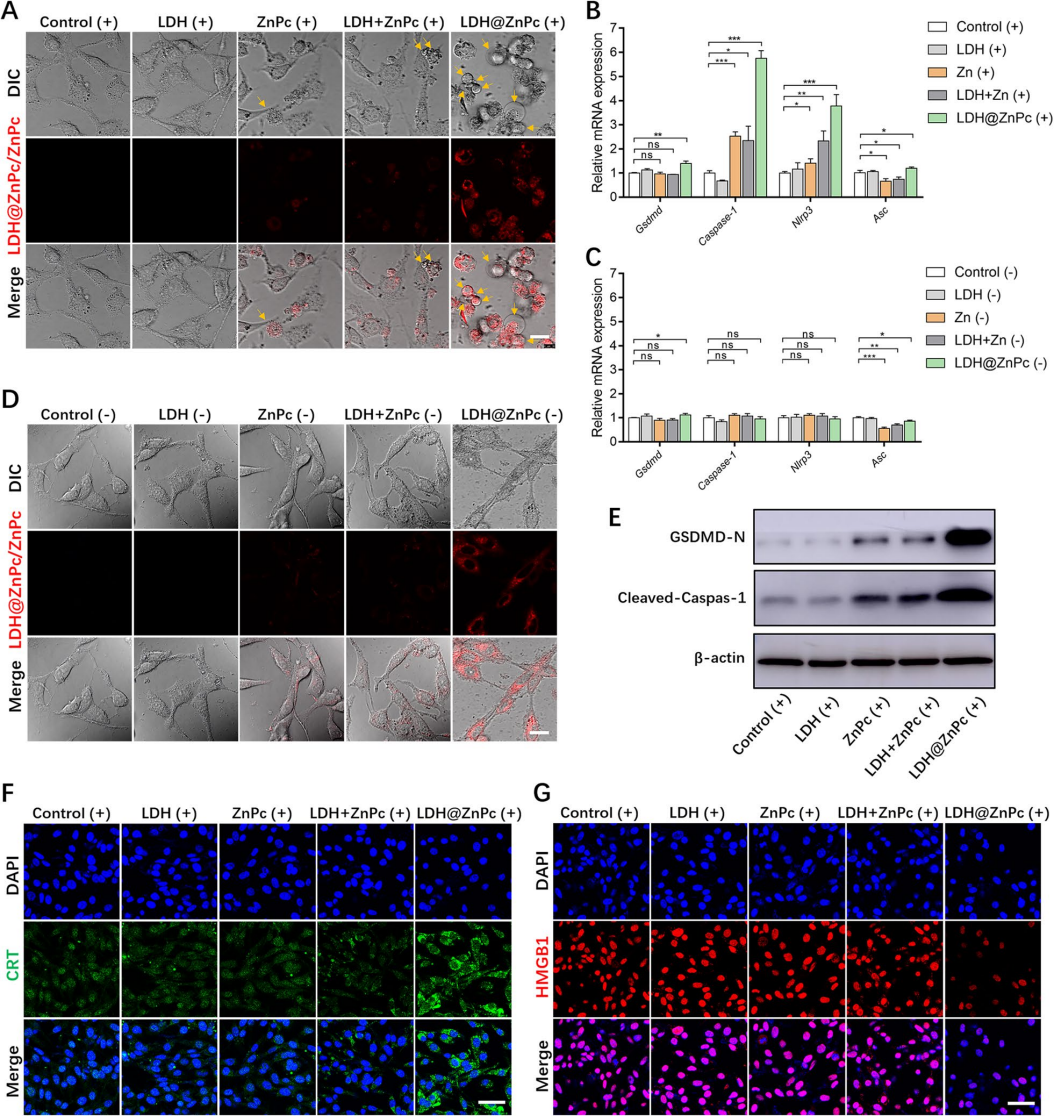
**A** Cell images of various groups after irradiation from LED light (670 nm, 3 J cm^−2^). Scale bars: 20 µm. **B**, **C** Relative mRNA expression levels of *Gsdmd*, *Caspase-1*, *Nlrp3* and *Asc* in various groups after irradiation from LED light (670 nm, 3 J cm^−2^) (**B)** and in the dark (**C**). Data are expressed as means ± s.d. (n = 5). *p < 0.05, **p < 0.01, ***p < 0.001, ****p < 0.0001. **D** Cell images of various groups in the dark. Scale bars: 20 µm. **E** Protein expression of GSDMD-N, Cleaved-Caspase-1 in different groups after irradiation from LED light (670 nm, 3 J cm^−2^). **F**, **G** CLSM images displaying the CRT (green fluorescence) and HMGB1 (red fluorescence) expression in TC-1 cells after incubation with LDH@ZnPc and subsequent light irradiation from LED light (670 nm, 3 J cm^−2^). The cell nuclei were stained by DAPI (blue fluorescence). Scale bars: 50 µm

As reflected in Fig. 3A, cells treated with PBS and LDH upon irradiation showed a relatively intact cell membrane without obvious morphological changes. Upon treatment with ZnPc or a mixture of LDH and ZnPc under the same irradiation, only a minor quantity of cells swelled with multiple small bubble-like protrusions (as indicated by yellow arrows) around cell membranes. In contrast, for the group incubated with LDH@ZnPc, an increased number of cells swelled and blew out large or small bubbles (as indicated by yellow arrows) from the plasma membrane. Furthermore, in the absence of light, the cell morphology of each group remained unchanged (Fig. 3D). Interestingly, this classic bubble-like cell morphology is considered as the typical characteristic of pyroptosis, a newly disclosed manner of programmed cell death. Therefore, the cell morphological change of group LDH@ZnPc may indicate the occurrence of pyroptosis.

According to recent research, the intracellular ROS was identified to active NOD-like receptor family pyrin domain protein 3 inflammasome (NLRP3), which subsequently recruit the adaptor protein apoptosis associated speck-like protein containing a caspase recruitment domain (ASC) to further recruit proCaspase-1, ultimately forming of inflammasome complex [17]. Caspase-1 is then activated within the inflammasome, and active Caspase-1 processes gasdermin D (GSDMD) to form its N-terminal domain (GSDMD-N), which is known as the canonical pyroptosis pathway. Therefore, to further ascertain the occurrence of pyroptosis and its mechanism after LDH@ZnPc-mediated PDT, we performed quantitative real-time PCR (qPCR) and western blot assay. In addition to *Asc*, the mRNA expression of *Gsdmd*, *Caspase-1*, and *Nlrp3* was not significantly changed in the absence of light (Fig. 3C), while under light conditions (Fig. 3B), LDH@ZnPc-treated groups showed significantly higher expressions of *Gsdmd*, *Caspases-1*, *Nlrp3* and *Asc* compared to control groups. Additionally, we noticed that the ZnPc and LDH + ZnPc reduced *Asc* expression at the mRNA level more significantly than LDH@ZnPc without light, which may be possible reason for the failure of ZnPc and LDH + ZnPc to increase *Asc* obviously when irradiated. More importantly, western blot analysis indicated that GSDMD-N and Cleaved-Caspase-1 levels were significantly increased in the LDH@ZnPc-treated group upon irradiation (Fig. 3E), providing the strongest evidence for the occurrence of canonical GSDMD-dependent pyroptosis.

### Evaluation of ICD efficacy

Previous studies have confirmed the occurrence of ICD can release DAMPs, such as calreticulin (CRT) and high-mobility group box 1 (HMGB1), which then evoke antitumor immune response systematically. CRT can act as an “eat me” signal when translocated to the cell surface, which further leads to the uptake of dying tumor cells and their debris by antigen presenting cells (APCs). HMGB1 as a chromatin-binding protein can be released from the nucleus in the advanced stages during cell death. In general, CRT exposure on the cancer cell surface and HMGB1 release from the nucleus were regarded as emblematic hallmarks indicating the generation of ICD. Pyroptosis was recently demonstrated as a novel type of ICD, which can induce antigens release and subsequently robust antigen specific immune responses. Moreover, the photosensitizer with excellent mitochondria-anchored ability evokes prominent mitochondria oxidative stress, and consequently induces ICD. Therefore, we investigated whether our photosensitizer could massively evoke ICD generation using CLSM. As shown in Fig. 3F, the LDH@ZnPc treated group displayed apparent surface CRT exposure, whereas the other groups failed to exhibit such a phenomenon. Meanwhile, the ability of HMGB1 release was also investigated, which indicated that the LDH@ZnPc treated group revealed significant decrease of fluorescence in the nucleus, but no obvious changes were observed for the other groups (Fig. 3G). Taken together, these results verified the robust ICD generation triggered by pyroptosis and mitochondria anchoring in TC-1 cells after LDH@ZnPc-mediated PDT.

### In vivo fluorescence imaging and antitumor activity

**Fig. 4:**
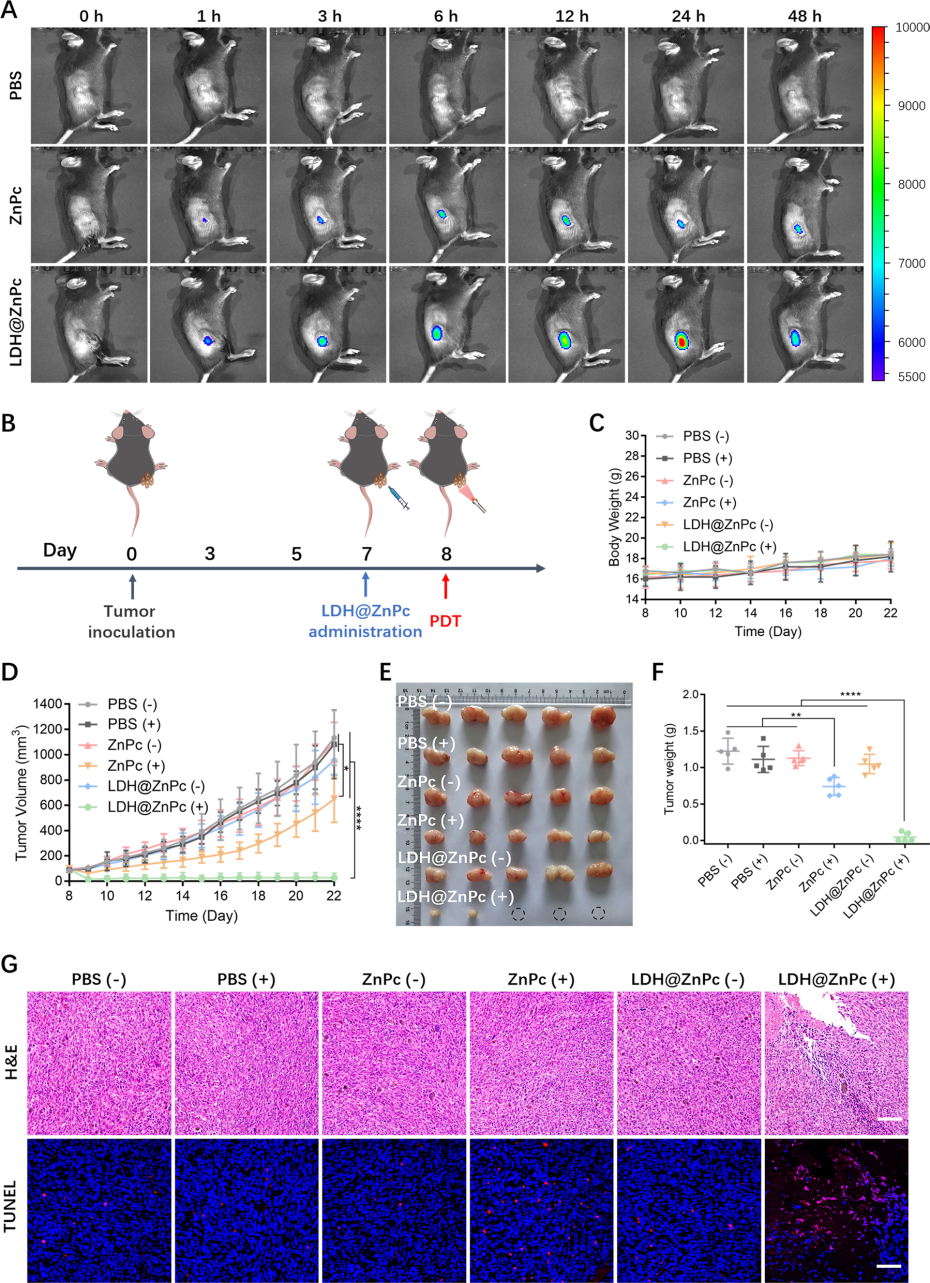
**A** In vivo fluorescence imaging of different groups using C57BL/6 mice bearing TC-1 tumors at various times. **B** Schematic illustration of TC-1 tumor model and the administration schedule. **C** Change curves of body weights of different groups within investigated days. **D** Change curves of tumor volume versus time. **E**, **F** Dissociated tumor image after 22 days (the dotted circles indicated the cured tumors) and excised tumor weights. **G** H&E and TUNEL staining of tumor tissues in various groups after light irradiation. Scale bars: 50 µm. All tumors were irradiated once using a laser light at 660 nm with a light dose of 120 J cm^−2^ (light power: 200 mW cm^−2^). Data are expressed as means ± s.d. (n = 5). *p < 0.05, **p < 0.01, ****p < 0.0001. (“ + ” and “−” denote with and without irradiation, respectively)

To investigate the retention and metabolism of LDH@ZnPc in tumor, the fluorescence imaging was conducted before in vivo antitumor experiments. As reflected in Fig. 4A, after intratumorally injecting the LDH@ZnPc, fluorescence intensity gradually increased and reached a maximum around 24 h in living mice. In contrast to LDH@ZnPc, ZnPc reached a maximum within 6–12 h. Additionally, LDH@ZnPc is more permeable to tumor tissue and can infiltrate the entire tumor tissue. Even 48 h after injection, there was an obvious fluorescence signal on the tumor tissue of LDH@ZnPc-treated group but not that of ZnPc-treated group, which indicated an ameliorative tumor retention ability when ZnPc was incorporated onto LDH.

An excellent photosensitizer should possess potent in vivo antitumor ability, we thus investigated the antitumor efficacy of LDH@ZnPc on C57BL/6 mice inoculated with TC-1 tumors. PBS- and ZnPc-treated groups with light irradiation were used as positive control, meanwhile groups of that without light irradiation were provided as negative reference. The light-treated groups were given a light dose of 120 J cm^−2^ from a 660 nm laser. To demonstrate the potent efficacy of LDH@ZnPc-mediated PDT, only single-time of irradiation was performed in irradiation-treated groups (Fig. 4B). As illustrated in Fig. 4D, E, LDH@ZnPc-treated group exhibited significant retardation of established TC-1 tumors in 3 of 5 mice (60%) and no trends of bounce was observed within investigated period. The weights of tumors were provided accordingly in Fig. 4F. ZnPc-treated group displayed certain inhibition against tumor growth, however, it was so poor that the tumors still rebounded on 9th day. As for the other groups, they displayed a similar trend of soared tumor growth without obvious inhibitory effects. Furthermore, data of body weight changes suggested that all the groups exhibited certain increase (Fig. 4C), indicating the low toxicity of LDH@ZnPc.

As depicted in Fig. 4G, hematoxylin and eosin (H&E) staining demonstrated that LDH@ZnPc-treated group exhibited severe destruction of tumor tissues with obvious spaces in comparison with other groups, which further verified its robust antitumor effect. Meanwhile, TUNEL staining was also performed with potent fluorescent intensity for LDH@ZnPc-treated group and certain intensity for ZnPc-treated group, which correlated well with their in vivo PDT antitumor effect. In addition, indexes of liver and kidney function revealed the security of various treatments (Additional file 1: Table S1). Besides, H&E staining of major organs (Additional file 1: Figure S6) confirmed the low toxicity of LDH@ZnPc. Collectively, these results manifested the robust in vivo antitumor efficacy and security of LDH@ZnPc-mediated PDT.

### Primary and distant tumor inhibition

**Fig. 5:**
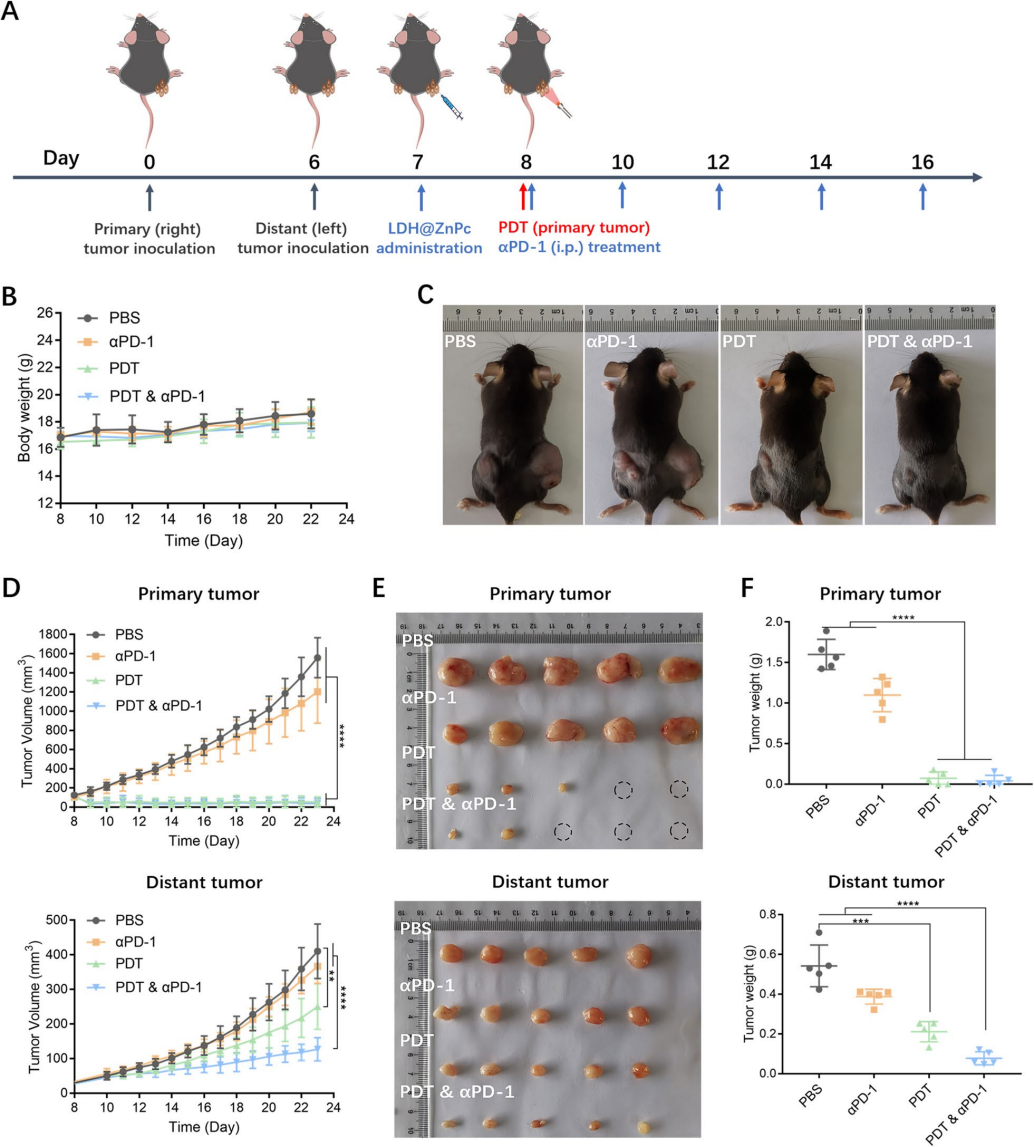
**A** Schematic illustration of a bilateral TC-1 tumor model and the administration schedule. **B** Change curves of body weight. **C** Representative photographs of mice bearing TC-1 tumors after various treatments. **D** Plot of tumor volume toward primary and distant tumors within investigated days. **E**, **F** Images of excised tumor after 23 days (the dotted circles represent cured tumors) of primary and distant tumors on a TC-1 bilateral tumor model and the corresponding weights of tumors. Data are expressed as means ± s.d. (n = 5). **p < 0.01, ***p < 0.001, ****p < 0.0001

Encouraged by these obtained results, we then investigated whether LDH@ZnPc-mediated PDT could work effectively towards primary and distant tumors. This assay was performed using a bilateral model of TC-1 tumors on C57BL/6 mice. The tumors on the right side (primary) were treated with LDH@ZnPc and irradiated, while the left side tumors (distant) were untreated (Fig. 5A). To well illustrate the immunity efficacy induced by PDT, group underwent combined therapy of LDH@ZnPc-mediated PDT and αPD-1 (PDT & αPD-1) was also investigated as reference. Besides, the mice treated with PBS or αPD-1 served as controls. The therapeutic efficacies of various treated regimens were evaluated by the growth volumes of both primary and distant tumors.

As revealed in Fig. 5C–F, both PDT and combined therapeutic groups significantly inhibited the tumor growth, which exhibited tumor growth inhibitory of 40% and 60%, respectively. The αPD-1 group displayed certain inhibition against tumor growth, however, it was so poor that the tumor growth rates still exhibited a similar trend as the PBS group. For the effect towards untreated distant tumors, groups of PBS or αPD-1 failed to show inhibitory effect with tumor growth rates of similar trend. To our delight, group of single LDH@ZnPc-mediated PDT displayed effective inhibition towards distant tumor (*p* < 0.01 compared with the PBS group). Furthermore, PDT & αPD-1 group displayed the most potent abscopal antitumor effect, which can well demonstrate that LDH@ZnPc has a significantly synergistic effect compared with group of single αPD-1. Besides, the body weights were steady with slight increment, indicating no systemic toxicity (Fig. 5B). Overall, the in vivo antitumor results manifested that LDH@ZnPc-mediated PDT can not only kill primary tumors, but also exhibited apparent inhibition toward abscopal tumors.

### PDT-induced pyroptosis in primary tumor

To further investigate the death patterns of primary tumor and immune state of distant tumor, the bilateral tumor tissues were conducted for transcriptomic analysis after treatment with PBS, αPD-1, PDT and PDT & αPD-1. In all, 1835 genes of primary tumor and 2007 genes of distant tumor were analyzed, and the genes expression was displayed by a hierarchically clustered heatmap (Additional file 1: Figure S7A, S8A). In addition, the distributions of differentially expressed genes (DEGs, fold change  > 1 and *p* < 0.05) are illustrated via a standard volcano plot. Besides, the DEGs in the PBS vs PDT-treated groups were also performed using Kyoto encyclopedia of genes and genomes (KEGG) pathway enrichment analysis as well as gene ontology (GO) enrichment analysis.

**Fig. 6:**
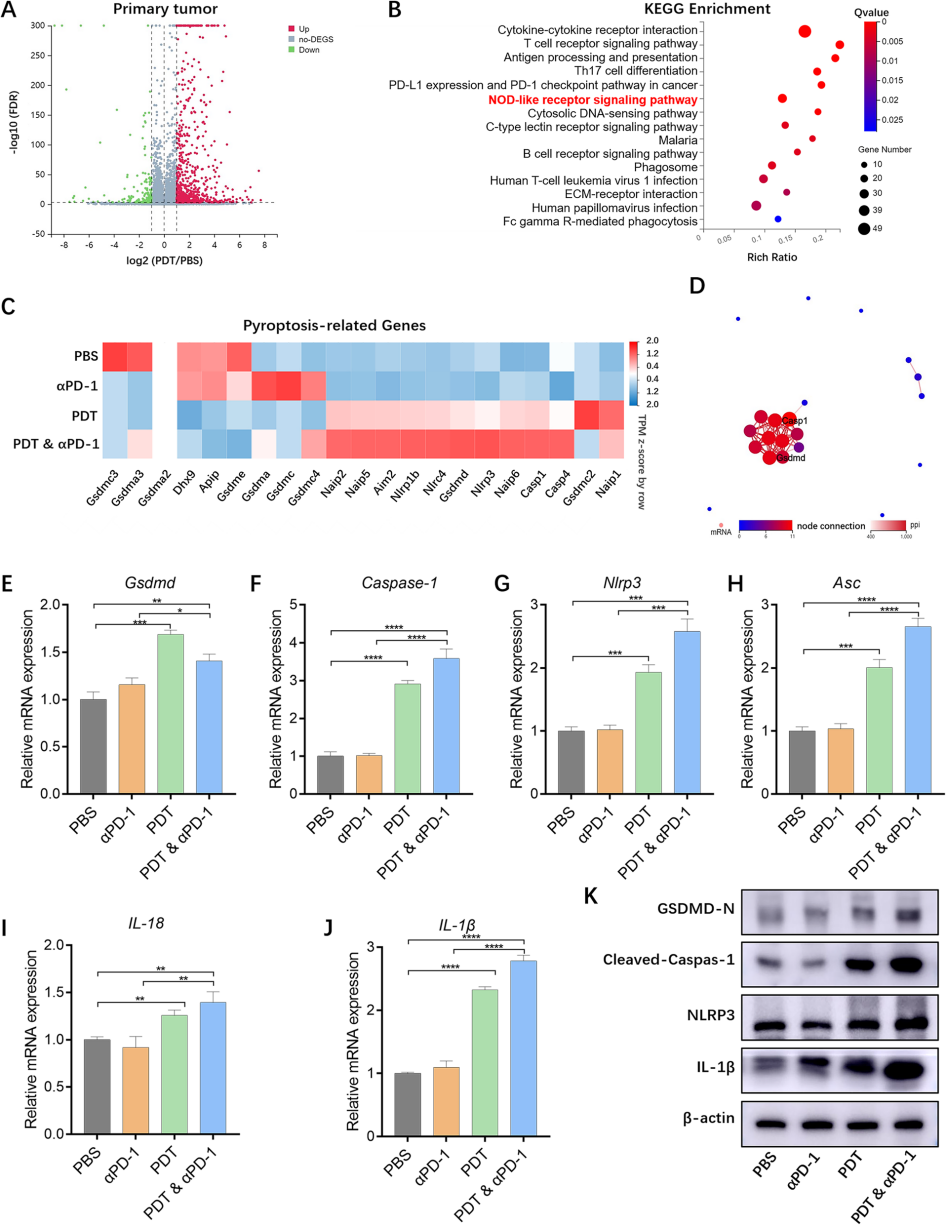
Transcriptomic analysis and PDT-induced pyroptosis of primary tumor. **A** Volcano plot of DEGs in the PBS vs PDT. **B** KEGG analyses of DEGs in the PBS vs PDT. **C** Heatmap of the pyroptosis-related gene expression based on transcriptomic analysis data of primary tumor. TPM is an expression measure normalized to sequencing depth. Genes that did not significantly changed in each comparison are colored white. **D** PPI network analysis of pyroptosis-related genes. **E**–**J** Verification of the expression of *Gsdmd* (**E**)*, Caspase-1* (**F**)*, Nlrp3* (**G**), *Asc* (**H**)*, IL-18* (**I**) and *IL-1β* (**J**) at the mRNA level. **K** Protein expression of GSDMD-N, Cleaved-Caspase-1, NLRP3, and IL-1β in different groups. Data are expressed as means ± s.d. (n = 5). *p < 0.05, **p < 0.01, ***p < 0.001, ****p < 0.0001

For primary tumor, a total of 897 DEGs were screened. As shown in Fig. 6A, the volcano plot showed that 730 genes were up-regulated and 167 genes were down-regulated after treatment with PDT. As identified by GO analysis of these DEGs (Additional file 1: Figure S7B), KEGG pathway proteins were enriched (Fig. 6B), including cytokine-cytokine receptor interaction, cell adhesion molecules, T cell receptor signaling pathway, natural killer cell mediated cytotoxicity, and NOD-like receptor signaling pathway (Additional file 1: Figure S7C). Recent studies have manifested that intracellularly generated ROS can trigger the pyroptosis, which involves the activation of multiple inflammasome signaling pathways including NOD-like receptor family pyrin domain protein 3 inflammasome (NLRP3) [17]. In our study, we have ascertained the occurrence of GSDMD-dependent pyroptosis after LDH@ZnPc-mediated PDT in vitro (Fig. 3A–E). To further confirm the occurrence of pyroptosis after LDH@ZnPc-mediated PDT in vivo, DEGs related to pyroptosis were screened on KEGG enrichment analyses. The results indicated that PDT increased levels of DEGs encoding proteins, such as gasdermin family proteins (GSDMD), cysteine aspartic acid-specific protease (Caspase-1), inflammasome (NLRP3), interleukin-1β (IL-1β), and interleukin-18 (IL-18), all of which are associated with pyroptosis (Fig. 6C, D). Furthermore, we examined the expression levels of these DEGs via qPCR, and the results were coincided with that obtained by transcriptomic analysis (Fig. 6E–J). Accordingly, the results of western assay also demonstrated the enhanced expression levels of GSDMD-N, Cleaved-Caspase-1, NLRP3, and IL-1β (Fig. 6K). Taken together, these data as well as cellular results jointly indicated that LDH@ZnPc-mediated PDT activated the canonical inflammasome pathway of GSDMD-dependent pyroptosis.

### Immune response in vivo

**Fig. 7:**
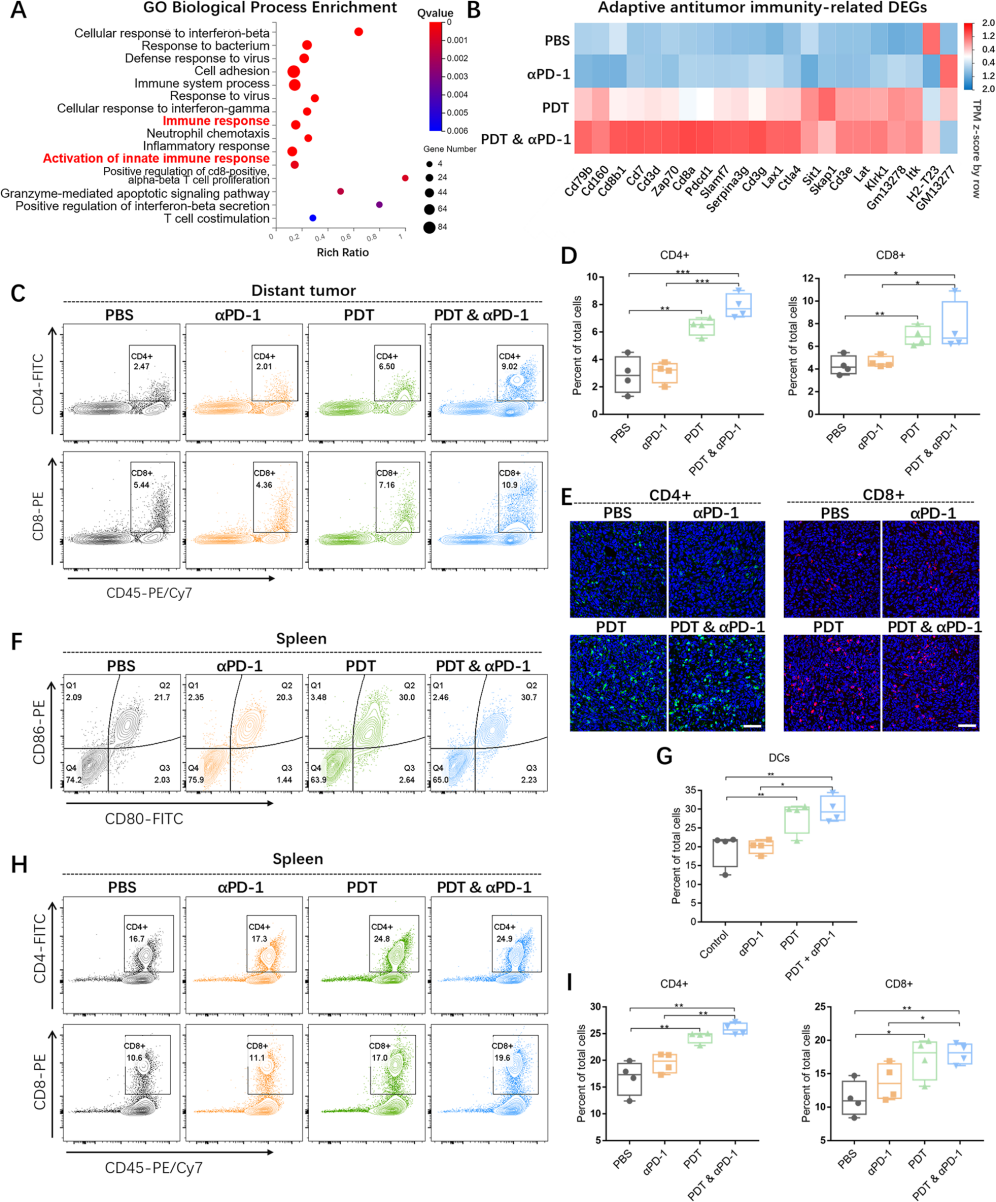
Transcriptomic analysis of distant tumor and immune response in vivo. **A** GO enrichment analyses of DEGs in the PBS vs PDT. **B** Adaptive antitumor immunity-related DEGs screened based on GO biological process enrichment analysis of distant tumor. **C**–**D** Evaluation of CD4 + and CD8 + T cells in distant tumors (n = 4). **E** Immunofluorescence images of CD4 + and CD8 + T cells in distant tumor tissues. Green and red fluorescence indicate CD4 + T cells and CD8 + T cells, respectively. Scale bars: 50 μm. **F**–**I** Expression of DCs (**F**, **G**), CD4 + T cells and CD8 + T cells (**H**, **I**) in spleen after different treatments detected by flow cytometry in vivo (n = 4)

To further investigate the immune state of distant tumor, DEGs of 483 up-regulated and 734 down-regulated were screened (Additional file 1: Figure S8B) with a result of significant difference in gene expression between PBS and PDT groups. Subsequently, the DEGs were analyzed by GO enrichment and KEGG pathway enrichment analyses (Fig. 7A, Additional file 1: Figure S8C). In particular, the differentially expressed genes related to “immune response” and “adaptive antitumor immunity” were screened to evaluate the antitumor immune effect of LDH@ZnPc-mediated PDT (Fig. 7B, Additional file 1: Figure S8D). Compared with the PBS group, αPD-1 failed to obviously up-regulate these immune-related genes, suggesting an occurrence of poor immune response. On the contrary, the distant tumor in PDT group displayed an increased up-regulation of these immune-related genes, indicating the effective immune activation triggered by LDH@ZnPc-mediated PDT.

To well examine the immune response in vivo, we further investigated the immune-related cell populations in distant tumors as well as spleens at day 15 after various treatments. TC-1 tumor-bearing mice were euthanized to obtain the tumor and spleen tissues, which were then performed for flow cytometry analysis. As illustrated in Fig. 7C, the percentages of CD4 + and CD8 + T lymphocytes in the distant tumors were significantly increased in PDT-treated groups compared with other groups. Concretely, single LDH@ZnPc-mediated PDT and PDT&αPD-1 group exhibited cell percentages of 6.40% ± 0.32% and 7.87% ± 0.43% respectively in terms of CD4 + T cells, which is ~ 2 − 3 folds compared with those of PBS and αPD-1 groups. As for CD8 + T cells, the data of PDT-treated groups still demonstrated similar differentiation in comparison with those of other groups (Fig. 7D). Meanwhile, the levels of CD4 + and CD8 + T cells in distant tumor tissues were further investigated using immunofluorescence staining (Fig. 7E), revealing that the fluorescence intensities in groups of PDT treatment were obviously stronger than the other groups, which is correlated well with the results of flow cytometry.

Moreover, the levels of immune cells in spleens were further investigated as well. As DCs was vital in initiating adaptive anti-tumor immunity, we therefore assessed the maturation of DCs in spleen. As displayed in Fig. 7F, G, the percentage of CD80 + CD86 + DCs in PDT and PDT&αPD-1 groups were 28.00% ± 2.14% and 29.93% ± 1.71% respectively, which is significantly increased compared with that of PBS (19.38% ± 2.30%) and αPD-1 group (20.03% ± 0.92%), suggesting the enhanced level of DCs maturation after LDH@ZnPc-mediated PDT treatment. As depicted in Fig. 7H, I, PDT-treated groups all demonstrated increased levels of CD4 + and CD8 + T cells compared with PBS and αPD-1 groups in spleen. In both distant tumor and spleen analysis, all PDT&αPD-1 groups obviously enhanced the blockade efficacy of αPD-1 therapy, which can be ascribed to the reason that LDH@ZnPc-mediated PDT elicited efficient ICD and further enhanced the efficacy of immune checkpoint blockade therapy.

Taken together, these results demonstrated all PDT-treated groups triggered potent immunological response. More importantly, LDH@ZnPc-mediated PDT significantly enhanced the efficacy of anti-PD-1 therapy, which consequently may act as a promising immune adjuvant to boost antitumor immunity.

## Conclusion

In summary, our work revealed that LDH@ZnPc can act as an excellent pyroptosis inducer with simultaneous mitochondria anchoring ability for enhancing photodynamic therapy and boosting antitumor immunity. Efforts to reduce aggregation of ZnPc was achieved by its efficient isolation using LDH without complicated preparation. LDH@ZnPc exhibited excellent mitochondria-anchored ability, which can massively evoke mitochondrial oxidative stress, and consequently enhanced ICD. A more interesting finding is that LDH@ZnPc-mediated PDT can induce GSDMD-dependent pyroptosis in tumor cells. Determination of CRT and HMGB1, desirable inhibitory effects toward distant tumors, as well as increased immune related cells demonstrated the potency of LDH@ZnPc-mediated antitumor immune efficacy. In vivo results verify potent tumor inhibition efficacy, and antitumor immunity towards distant metastatic tumor. Our work may encourage the scientific community, using simplified and convenient preparation, to discover imperative photosensitizers featured with potent PDT efficacy and desirable antitumor immunity.

## Supplementary Information

**Additional file 1: Figure S1.** Uptake determination of LDH@ZnPc and ZnPc at different incubated times. The cell nuclei were stained by DAPI (blue fluorescence). Scale bars: 50 *µ*m. **Figure S2.** Colocalization investigation of Endoplasmic reticulum-Tracker Green and Golgi apparatus-Tracker Green with LDH@ZnPc at 24 h. Scale bar: 20 *µ*m. **Figure S3. **Confocal fluorescence images reflecting the changes of mitochondria membrane potential in TC-1 cells using JC-1 dye as the indicator. Scale bar: 50 *µ*m. **Figure S4.** Flow cytometric analysis evaluating cell viability in TC-1 cells with various treatments. (“+” and “-” denote with and without irradiation, respectively). **Figure S5.** Cell viability determination of various groups in HeLa cells after irradiation from LED light (670 nm, 3 J cm^-2^) or in the dark. (“+” and “-” denote with and without irradiation, respectively). **Figure S6.** H&E staining of major organs in various groups after different treatments. For groups with irradiation, a laser light at 660 nm was used with a light dose of 120 J cm^-2^ (light power: 200 mW cm^-2^. “+” and “-” denote with and without irradiation, respectively). Scale bars: 50 *µ*m. **Figure S7.** Transcriptomic analysis of primary tumor. (A) Hierarchically clustered heatmap of DEGs. (B) GO enrichment analyses of DEGs in the PBS vs PDT. (C) NOD-like receptor signaling-related DEGs screened based on KEGG enrichment analyses of primary tumor. **Figure S8.** Transcriptomic analysis of distant tumor. (A) Hierarchically clustered heatmap of DEGs. (B) Volcano plot of DEGs in the PBS vs PDT. (C) KEGG analyses of DEGs in the PBS vs PDT. (D) Immune response-related DEGs screened based on GO biological process enrichment analysis of distant tumor. **Table S1.** Liver and kidney functional indexes of mice in each experimental group after different treatments. **Table S2. **Sequences of primers used for qPCR detection.

## Data Availability

The raw data and processed data required to reproduce these findings are available from the corresponding author upon request.
