# Supplementary material for: A mitochondria-anchored supramolecular photosensitizer as a pyroptosis inducer for potent photodynamic therapy and enhanced antitumor immunity
Source: J Nanobiotechnology. 2022 Dec 3;20:513. doi: 10.1186/s12951-022-01719-9 (PMC9719646; doi:10.1186/s12951-022-01719-9)
Supplement: Supplementary file 1 — Additional file 1: Figure S1. Uptake determination of LDH@ZnPc and ZnPc at different incubated times. The cell nuclei were stained by DAPI (blue fluorescence). Scale bars: 50 µm. Figure S2. Colocalization investigation of Endoplasmic reticulum-Tracker Green and Golgi apparatus-Tracker Green with LDH@ZnPc at 24 h. Scale bar: 20 µm. Figure S3. Confocal fluorescence images reflecting the changes of mitochondria membrane potential in TC-1 cells using JC-1 dye as the indicator. Scale bar: 50 µm. Figure S4. Flow cytometric analysis evaluating cell viability in TC-1 cells with various treatments. (“+” and “-” denote with and without irradiation, respectively). Figure S5. Cell viability determination of various groups in HeLa cells after irradiation from LED light (670 nm, 3 J cm-2) or in the dark. (“+” and “-” denote with and without irradiation, respectively). Figure S6. H&E staining of major organs in various groups after different treatments. For groups with irradiation, a laser light at 660 nm was used with a light dose of 120 J cm-2 (light power: 200 mW cm-2. “+” and “-” denote with and without irradiation, respectively). Scale bars: 50 µm. Figure S7. Transcriptomic analysis of primary tumor. (A) Hierarchically clustered heatmap of DEGs. (B) GO enrichment analyses of DEGs in the PBS vs PDT. (C) NOD-like receptor signaling-related DEGs screened based on KEGG enrichment analyses of primary tumor. Figure S8. Transcriptomic analysis of distant tumor. (A) Hierarchically clustered heatmap of DEGs. (B) Volcano plot of DEGs in the PBS vs PDT. (C) KEGG analyses of DEGs in the PBS vs PDT. (D) Immune response-related DEGs screened based on GO biological process enrichment analysis of distant tumor. Table S1. Liver and kidney functional indexes of mice in each experimental group after different treatments. Table S2. Sequences of primers used for qPCR detection. [file 12951_2022_1719_MOESM1_ESM.docx]

**Supporting Information**

**A mitochondria-anchored supramolecular photosensitizer as a pyroptosis inducer for potent photodynamic therapy and enhanced antitumor immunity**

*Hong Wang* ^a^*, Guoxin Jing* ^a^*, Jintong Niu* ^a^*, Li Yang* ^a^*, Youyuan Li* ^a^*, Yi Gao* ^a^*, Huichao Wang* ^a^*,* *Xiaorong Xu* ^c^*, Yechang Qian* ^b^*^*^*, *and Shilong Wang* ^a^*^*^*

^a^ Research Center for Translational Medicine at East Hospital, School of Life Science and Technology, Tongji University, Shanghai 200092, P. R. China

^b^ Department of Respiratory Disease, Baoshan District Hospital of Integrated Traditional Chinese and Western Medicine, Shanghai 201900, P. R. China

^c^ Department of Gastroenterology, Shanghai Tenth People’s Hospital, School of Medicine, Tongji University, Shanghai, 200072, P. R. China

^*^Corresponding author

Yechang Qian (E-mail: qianyechang@163.com)

Shilong Wang (E-mail: wsl@tongji.edu.cn)

**
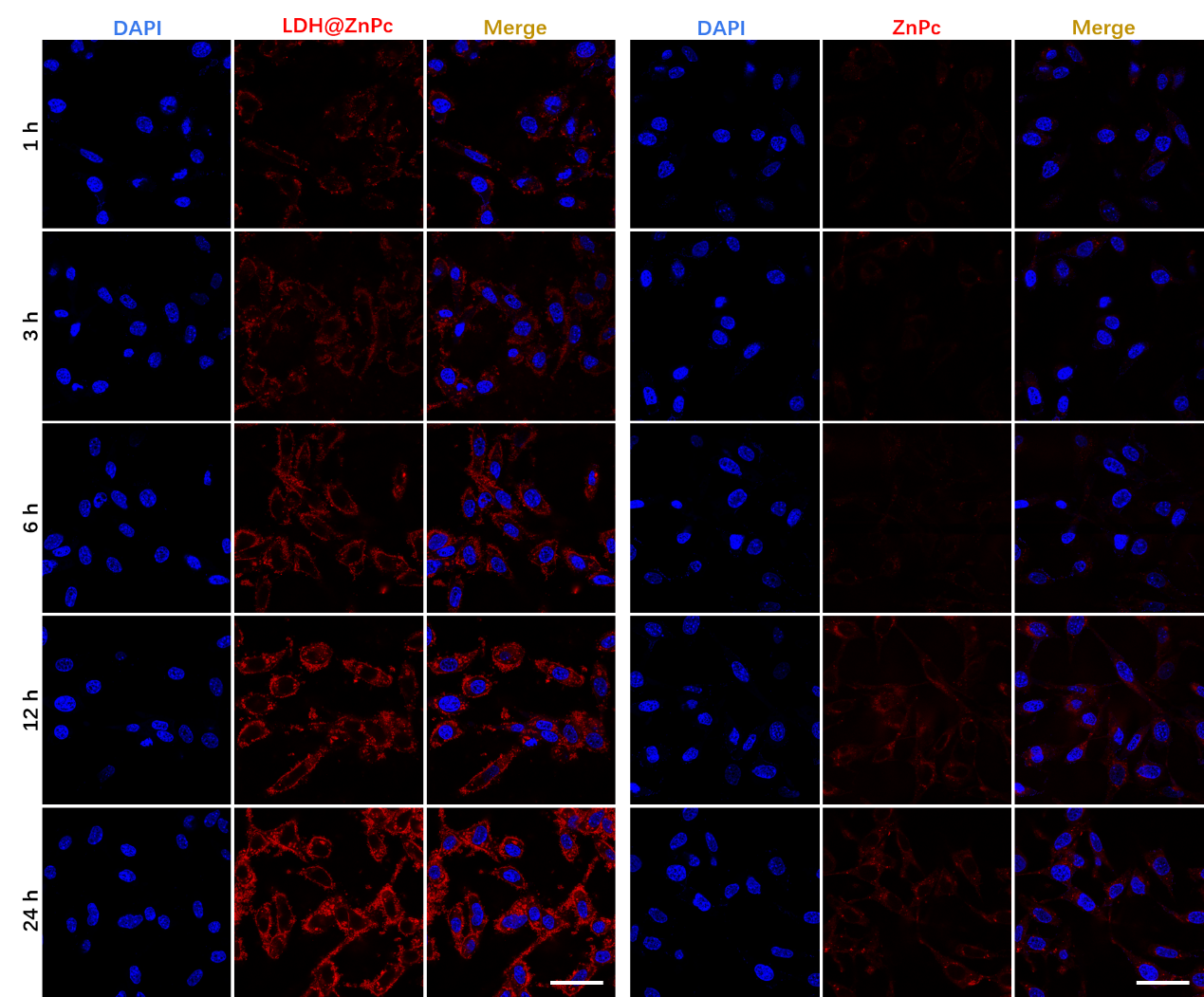
**

**Figure S1.** Uptake determination of LDH@ZnPc and ZnPc at different incubated times. The cell nuclei were stained by DAPI (blue fluorescence). Scale bars: 50 *µ*m.

**
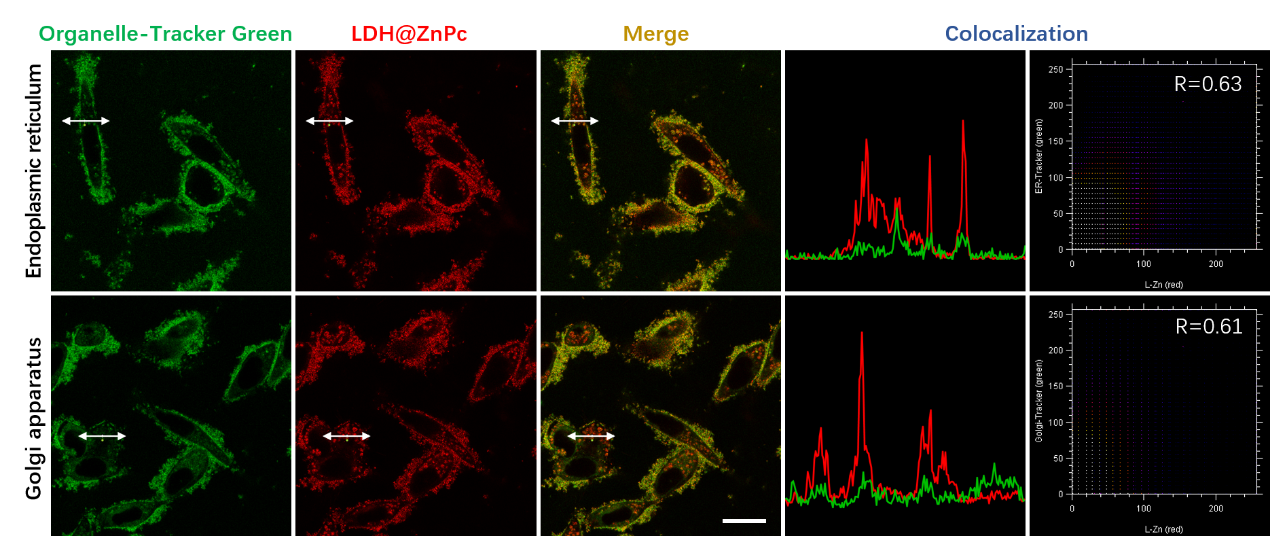
**

**Figure S2.** Colocalization investigation of Endoplasmic reticulum-Tracker Green and Golgi apparatus-Tracker Green with LDH@ZnPc at 24 h. Scale bar: 20 *µ*m.

**
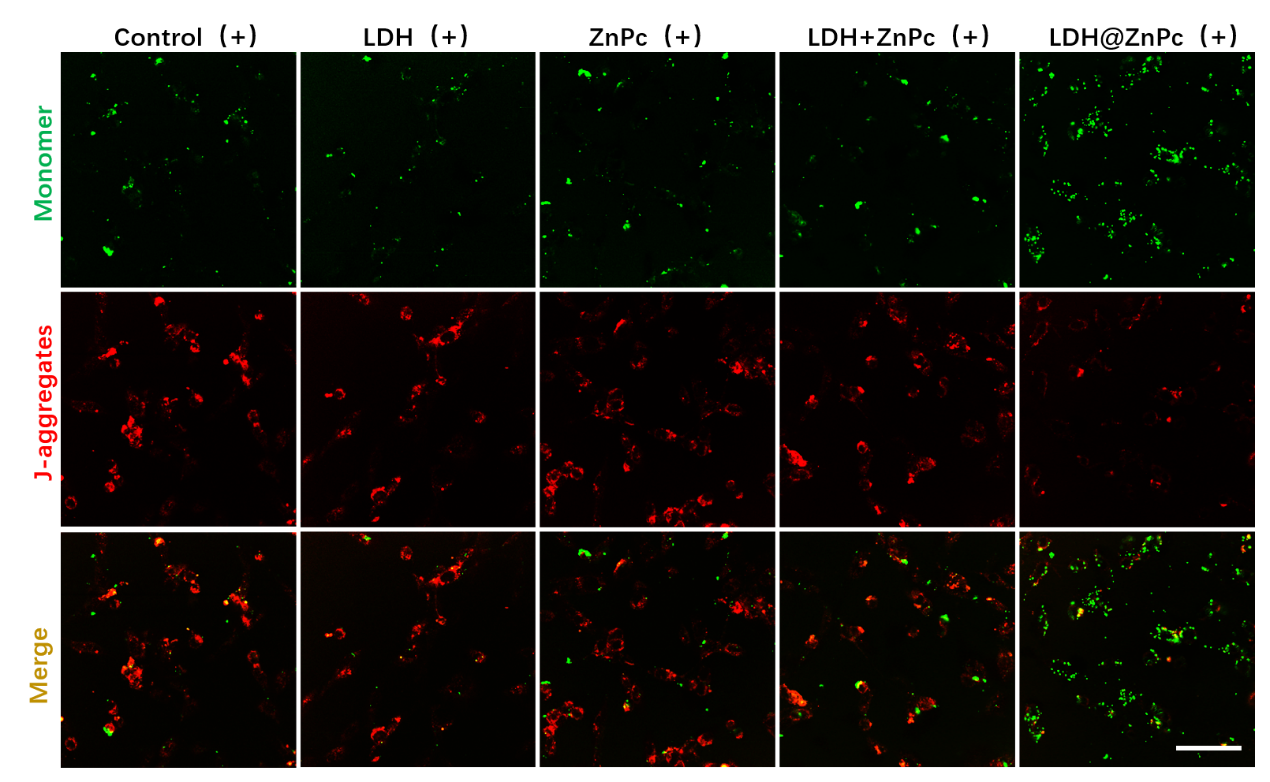
**

**Figure S3.** Confocal fluorescence images reflecting the changes of mitochondria membrane potential in TC-1 cells using JC-1 dye as the indicator. Scale bar: 50 *µ*m.

**
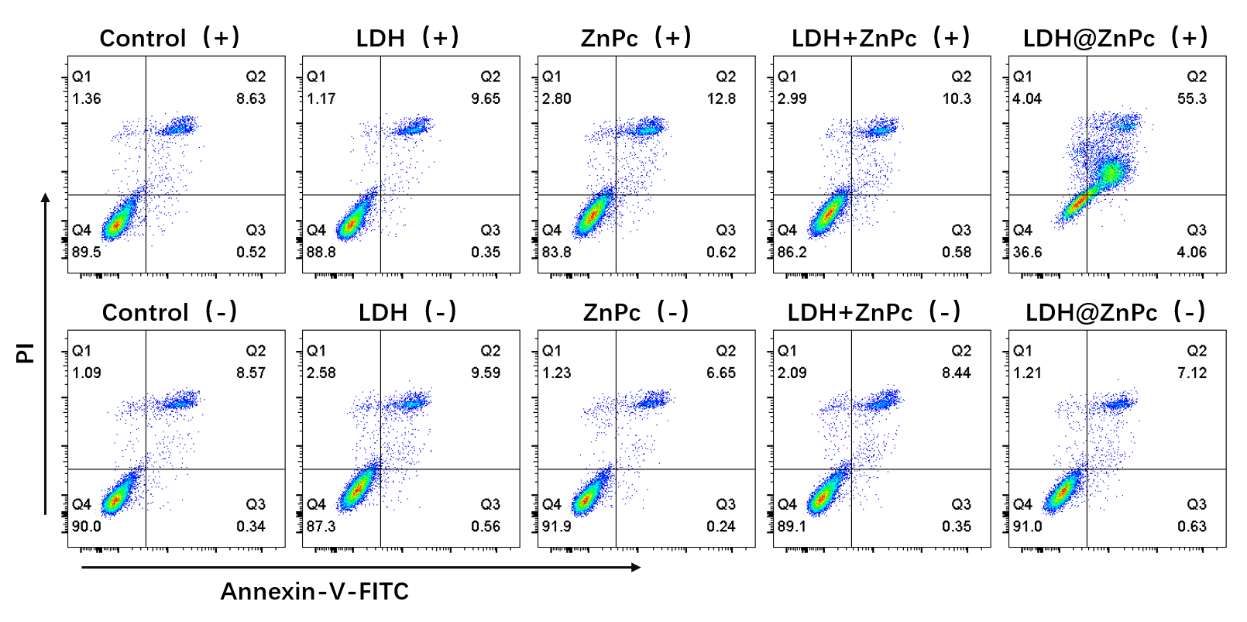
**

**Figure S4.** Flow cytometric analysis evaluating cell viability in TC-1 cells with various treatments. (“+” and “-” denote with and without irradiation, respectively)

**
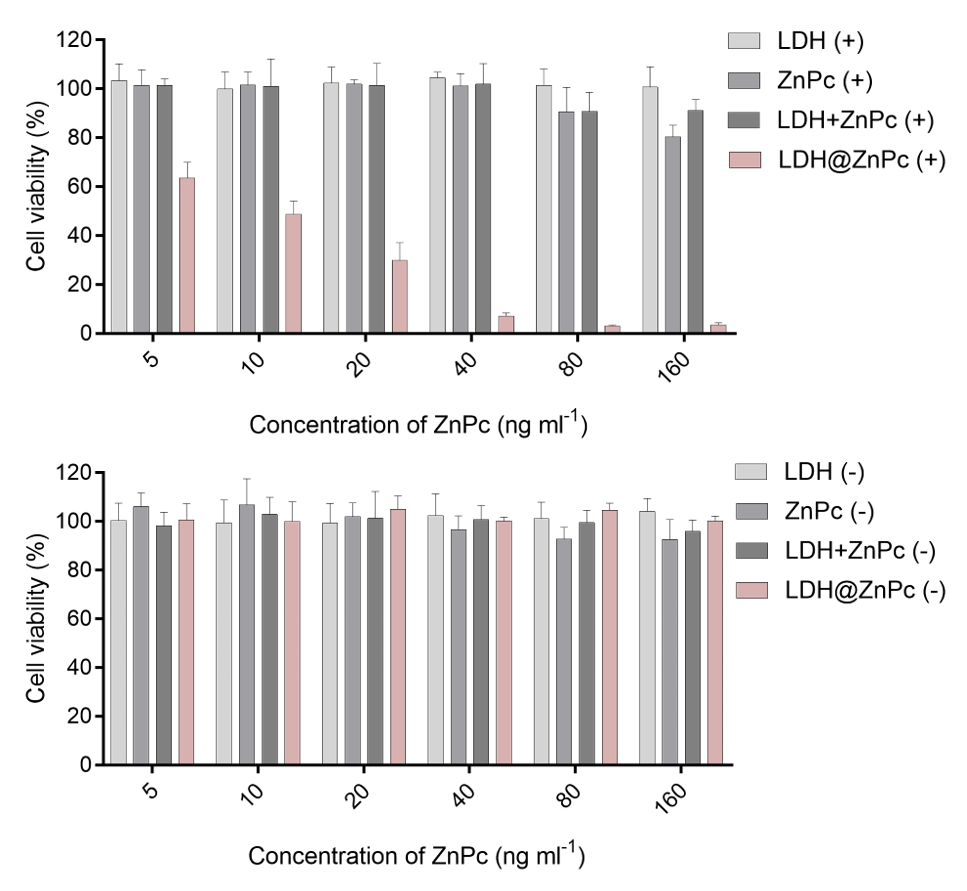
**

**Figure S5.** Cell viability determination of various groups in HeLa cells after irradiation from LED light (670 nm, 3 J cm^-2^) or in the dark. (“+” and “-” denote with and without irradiation, respectively)

**
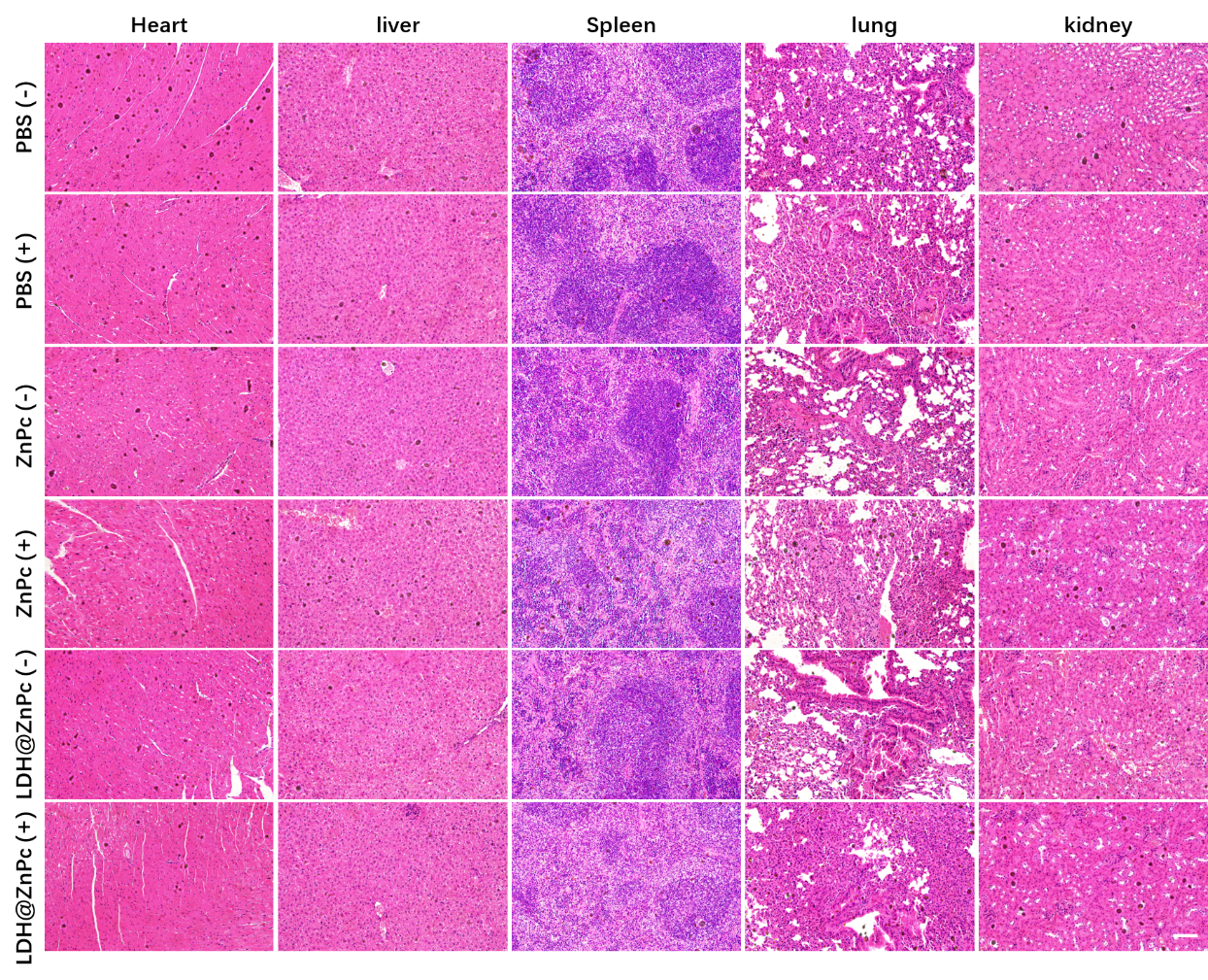
**

**Figure S6.** H&E staining of major organs in various groups after different treatments. For groups with irradiation, a laser light at 660 nm was used with a light dose of 120 J cm^-2^ (light power: 200 mW cm^-2^. “+” and “-” denote with and without irradiation, respectively). Scale bars: 50 *µ*m.

**
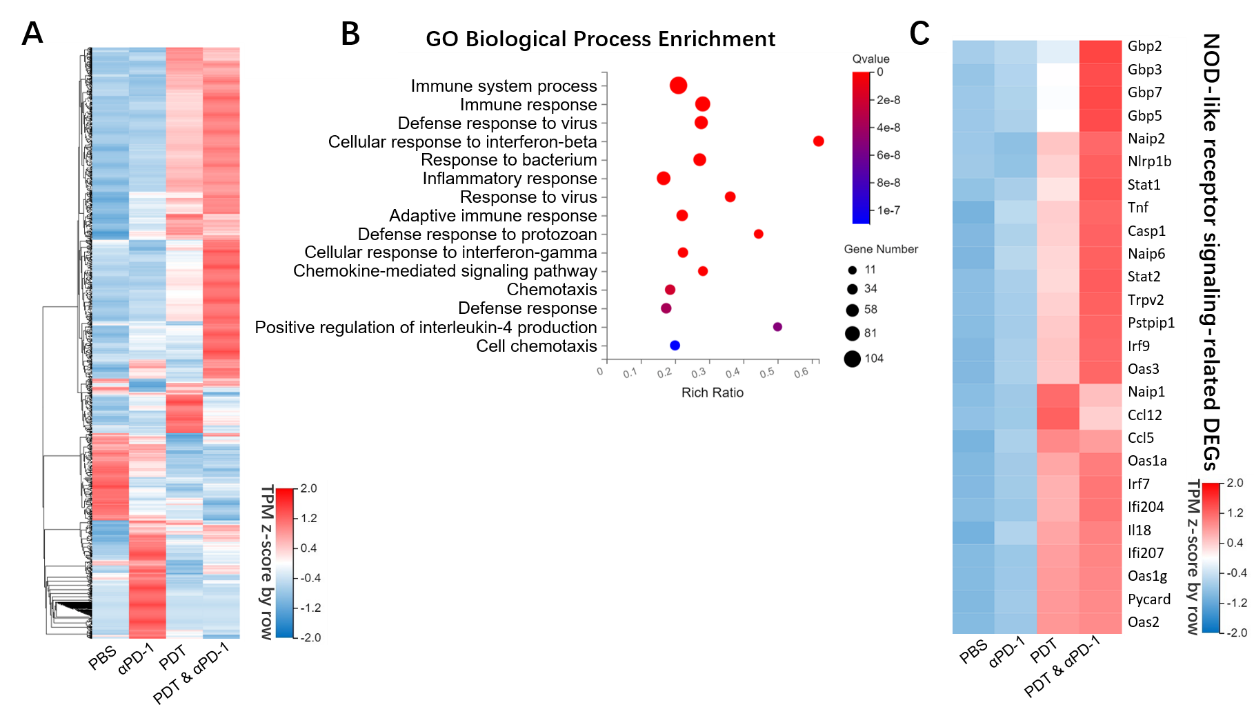
Figure S7.** Transcriptomic analysis of primary tumor. (A) Hierarchically clustered heatmap of DEGs. (B) GO enrichment analyses of DEGs in the PBS vs PDT. (C) NOD-like receptor signaling-related DEGs screened based on KEGG enrichment analyses of primary tumor.

**
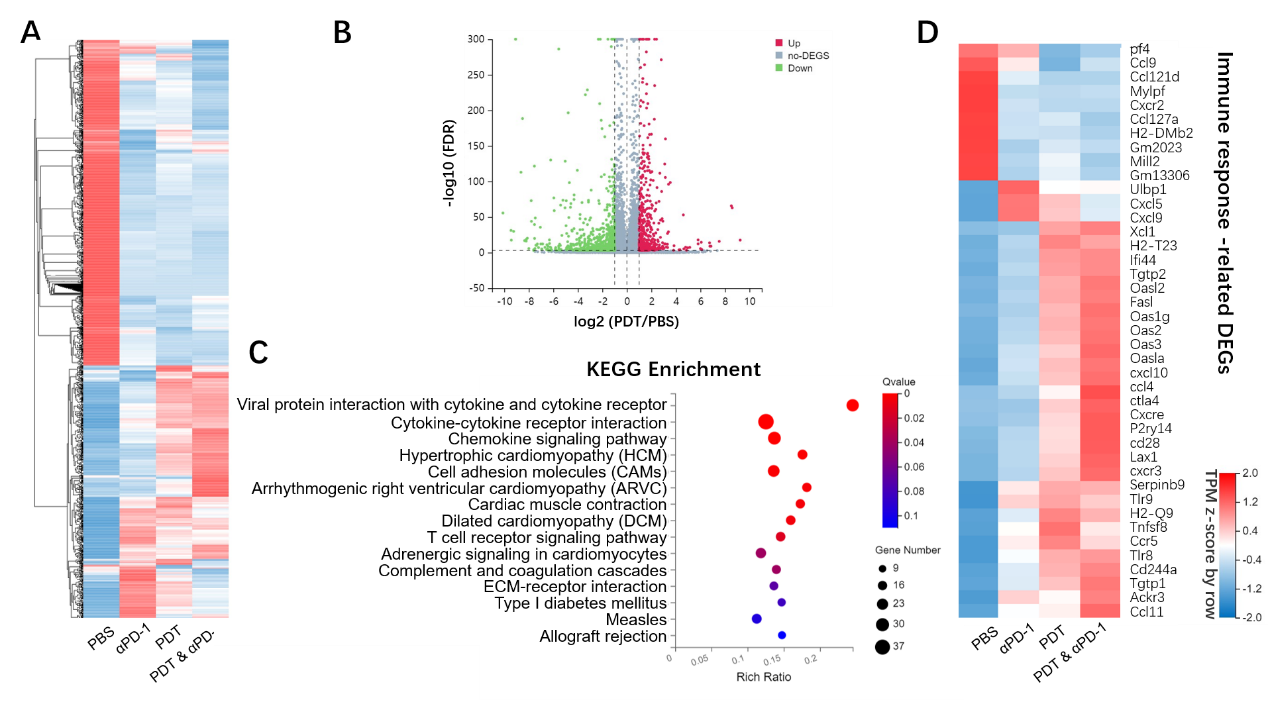
**

**Figure S8.** Transcriptomic analysis of distant tumor. (A) Hierarchically clustered heatmap of DEGs. (B) Volcano plot of DEGs in the PBS vs PDT. (C) KEGG analyses of DEGs in the PBS vs PDT. (D) Immune response-related DEGs screened based on GO biological process enrichment analysis of distant tumor.

**Table S1.** Liver and kidney functional indexes of mice in each experimental group after different treatments.

| **Sample** | **ALT**  **(U L^-1^)** | **AST**  **(U L^-1^)** | **BUN**  **(mg dL^-1^)** | **UA**  **(*μ*mol L^-1^)** | **CREA**  **(*μ*mol L^-1^)** |
| --- | --- | --- | --- | --- | --- |
| Ref range | 10.1-96.5 | 36.31-235.48 | 10.81-34.74 | 44.4-224.8 | 10.91-85.09 |
| PBS (-) | 32.771 | 188.511 | 19.656 | 122.242 | 20.912 |
| PBS (+) | 38.501 | 142.504 | 31.724 | 122.242 | 21.072 |
| ZnPc (-) | 35.469 | 91.450 | 31.108 | 91.056 | 17.041 |
| ZnPc (+) | 40.712 | 170.126 | 29.876 | 94.470 | 20.829 |
| LDH@ZnPc (-) | 38.581 | 134.819 | 41.160 | 105.169 | 19.035 |
| LDH@ZnPc (+) | 42.957 | 109.469 | 39.648 | 91.966 | 17.675 |

**Table S2.** Sequences of primers used for qPCR detection.

| Gene | Forward sequence (5’-3’) | Reverse sequence (5’-3’) |
| --- | --- | --- |
| *Gsdmd* | ATGCCATCGGCCTTTGAGAAA | AGGCTGTCCACCGGAATGA |
| *Caspase-1* | ACAAGGCACGGGACCTATG | TCCCAGTCAGTCCTGGAAATG |
| *Asc* | GACAGTGCAACTGCGAGAAG | CGACTCCAGATAGTAGCTGACAA |
| *Nlrp3* | ATTACCCGCCCGAGAAAGG | CATGAGTGTGGCTAGATCCAAG |
| *Il-18* | GTGAACCCCAGACCAGACTG | CCTGGAACACGTTTCTGAAAGA |
| *Il-1β* | GAAATGCCACCTTTTGACAGTG | TGGATGCTCTCATCAGGACAG |
| *Gapdh* | AGGTCGGTGTGAACGGATTTG | GGGGTCGTTGATGGCAACA |
